# Inhibition of BRD4 sensitizes NSCLC cells to osimertinib by suppressing APT1 and promoting MST1 palmitoylation

**DOI:** 10.1038/s41420-025-02794-1

**Published:** 2025-11-03

**Authors:** Shaoqiang Wang, Yingying Zheng, Zhenyu Zhang, Lijie Qiu, Wolong Zhou, Heng Zhang

**Affiliations:** 1 Department of Thoracic Surgery, Weifang People’s Hospital, Shandong Second Medical University, Weifang, Shandong Province P. R. China; 2 Department of Scientific Research Management, Weifang People’s Hospital, Shandong Second Medical University, Weifang, Shandong Province P. R. China; 3 Management Center, Weifang People’s Hospital, Shandong Second Medical University, Weifang, Shandong Province P. R. China; 4 Department of Respiratory Medicine, Weifang People’s Hospital, Shandong Second Medical University, Weifang, Shandong Province P. R. China; 5 Department of Thoracic Surgery, Xiangya Hospital, Central South University, Changsha, Hunan Province P. R. China; 6 Xiangya Lung Cancer Center, Xiangya Hospital, Central South University, Changsha, Hunan Province P. R. China

**Keywords:** Cancer, Cell biology

## Abstract

Osimertinib is widely used to treat non-small-cell lung cancer (NSCLC) carrying epidermal growth factor receptor (EGFR) mutations. However, osimertinib resistance inevitably develops in almost all patients. In our study, osimertinib-resistant HCC827/OR and PC-9/OR cells were established from parental osimertinib-sensitive cells, and osimertinib (AZD9291) and NHWD870, a bromodomain and extra-terminal (BET) inhibitor, were used to treat cells and mice. PC-9/OR and HCC827/OR cells were subcutaneously injected into mice to establish a mouse model of NSCLC. Luciferase, electrophoretic mobility shift assay (EMSA), and chromatin immunoprecipitation (ChIP) assays were applied to analyze transcription factors (TFs) binding to the APT1 promoter. MST1 palmitoylation was examined with acyl resin-assisted capture (Acyl-RAC) assays. The interaction of YAP1 and BRD4 was evaluated by co-immunoprecipitation (Co-IP) and GST-pull down assays. Our study showed that YAP1 was highly expressed, and its nuclear translocation was increased in osimertinib-resistant NSCLC cells, and silencing of YAP1 overcame osimertinib resistance. BRD4 was upregulated, and NHWD870 significantly reversed YAP1-mediated osimertinib resistance. Moreover, decreased MST1 palmitoylation at C699 was observed in NSCLC cells that are resistant to osimertinib. Furthermore, knockdown of APT1 reduced YAP1 nuclear translocation and APT1-mediated MST1 depalmitoylation restored osimertinib sensitivity. Inhibition of BRD4 blocked YAP1-mediated APT1 transcription in NSCLC cells. In addition, the BRD4 inhibitor disrupted MST1 depalmitoylation by APT1 and recovered osimertinib sensitivity. In vivo administration of NHWD870 enhanced NSCLC cell sensitivity to osimertinib. These findings indicate that inhibition of BRD4 enhances NSCLC cell sensitivity to osimertinib through the APT1-MST1-YAP1 axis.

**Figure:**
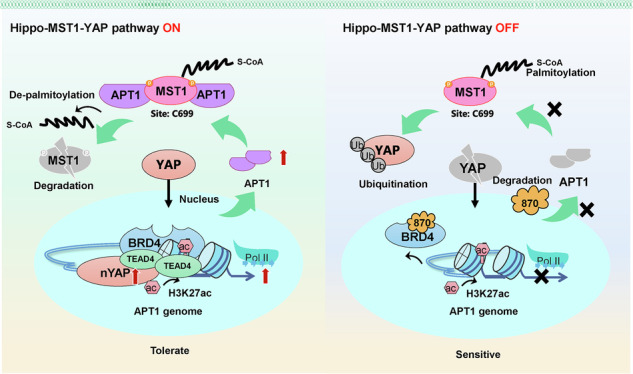
Inhibition of BRD4 sensitized non–small-cell lung cancer (NSCLC) cells to osimertinib by blocking YAP1-mediated APT1 transcription and disrupting APT1-mediated depalmitoyation of MST1 and YAP1 nuclear translocation, which restores osimertinib sensitivity through the APT1-MST1-YAP1 axis in NSCLC. Our study provides a novel mechanism of osimertinib resistance and suggests potential therapeutic strategies for NSCLC.

Inhibition of BRD4 sensitized non–small-cell lung cancer (NSCLC) cells to osimertinib by blocking YAP1-mediated APT1 transcription and disrupting APT1-mediated depalmitoyation of MST1 and YAP1 nuclear translocation, which restores osimertinib sensitivity through the APT1-MST1-YAP1 axis in NSCLC. Our study provides a novel mechanism of osimertinib resistance and suggests potential therapeutic strategies for NSCLC.

## Introduction

A large portion of non–small-cell lung cancer (NSCLC) harbors epidermal growth factor receptor (EGFR) mutations [1]. Exons 19 to 21 of the EGFR tyrosine kinase region are the major locations of mutations that may lead to overexpression of EGFR to contribute to the onset and progression of NSCLC [2, 3]. Tyrosine kinase inhibitors show beneficial anti-tumor effects for NSCLC containing EGFR mutations [4]. AZD9291 (Osimertinib) is a highly potent and selective EGFR tyrosine kinase inhibitor that irreversibly binds to the catalytically active center of EGFR and inhibits its activity, which is highly effective in advanced NSCLC treatment [5, 6]. However, patients develop osimertinib resistance after a median of 9.6 to 11 months, and the acquired osimertinib resistance significantly limits the anti-tumor effects in patients with NSCLC [7, 8]. Emerging evidence suggests that Yes-associated protein 1 (YAP1) is a key regulator of osimertinib resistance. YAP1 synergizes with dual specificity phosphatase 1 (DUSP1) to promote osimertinib resistance in NSCLC, and the YAP1/ TEA domain transcription factor (TEAD) axis has been identified as a driver of osimertinib resistance [9, 10]. However, the mechanisms involving YAP1 underlying osimertinib resistance have not been fully understood.

YAP1 acts as a transcriptional co-activator of the Hippo signaling that serves vital roles in cancer progression and drug resistance [11]. As a core component of the Hippo signaling pathway, mammalian sterile 20-like kinase 1 (MST1) regulates physiological processes such as cell differentiation, adhesion, and migration, and homeostasis, maintaining this through intranuclear and extranuclear localization, dimerization, and phosphorylation [12, 13]. Importantly, disruption of the Hippo/MST1 axis may result in the nuclear translocation of YAP/transcriptional coactivator with a PDZ-binding domain (TAZ), and subsequently, the YAP/TAZ bind to TEADs to regulate gene expression, thus promoting cell proliferation and inhibiting apoptosis [14]. MST1 phosphorylates large tumor suppressor kinase 1/2 (LATS1/2), subsequently phosphorylating YAP1 to sequester YAP1 in the cytoplasm [13]. The cytoplastic retention of YAP1 is vital to maintain osimertinib sensitivity. In osimertinib-sensitive NSCLC cells, YAP1 exists predominantly as phosphorylated YAP1 (p-YAP1) in the cytoplasm, whereas in osimertinib-resistant NSCLC cells, YAP1 undergoes dephosphorylation and is translocated to the nucleus [15]. YAP1 nuclear translocation is essential for the acquired osimertinib resistance, and blocking the nuclear translocation may be key to recovering osimertinib sensitivity in NSCLC.

Acyl-protein thioesterase 1 (APT1), also known as lysophospholipase1 (LYPLA1), has been identified as a palmitoyl thioesterase that depalmitoylates cysteine residues of proteins [16]. APT1 is upregulated in NSCLC cells, and inhibition of APT1 suppresses cell proliferation, migration, and invasion, supporting an oncogenic activity of APT1 [17], which also raises the possibility that APT1 promotes NSCLC progression via depalmitoylation. Intriguingly, several potential palmitoylation sites (C124, C369, and C699) of MST1 were predicted through bioinformatics, and we hypothesized that APT1 might depalmitoylate MST1 to regulate the Hippo signaling pathway and YAP1 nuclear translocation, thus inducing osimertinib resistance in NSCLC. However, it has never been reported.

As a transcriptional and epigenetic regulator, bromodomain protein 4 (BRD4) plays a key role in cancer initiation and development. In NSCLC, inhibition of BRD4 enhances tumor necrosis factor-related apoptosis-inducing ligand (TRAIL)-induced apoptosis and restrains tumor growth by downregulating eukaryotic translation initiation factor 4E (eIF4E) [18, 19]. Bromodomain and extra-terminal (BET) inhibitors, such as NHWD870, may lead to resistance to anticancer agents, revealing the effect of BRD4 in tumorigenesis and chemoresistance [20]. However, the linkage of BRD4 and osimertinib resistance in NSCLC remains unknown. A previous study has demonstrated that BRD4 interacts with YAP/TAZ to enhance gene transcription [21]. Additionally, it has been reported that a positive regulation of CCBE1 by YAP1/TAZ/BRD4 and their pro-tumor lymphangiogenesis in colorectal cancer [22]. Thus, BRD4 may be implicated in osimertinib resistance by interacting with YAP/TAZ in NSCLC. We propose the hypothesis that YAP1 interacts with BRD4 to recruit TEAD4 and activate APT1 transcription, and APT1-mediated MST1 depalmitoylation facilitates YAP1 nuclear translocation and promotes osimertinib resistance. Inhibition of BRD4 might inhibit APT1-mediated MST1 depalmitoylation to sequester YAP1 in the cytoplasm and promote osimertinib sensitivity.

Here, we demonstrated that silencing of YAP1 inhibited osimertinib resistance, and inhibition of BRD4 reversed YAP1-mediated osimertinib resistance in NSCLC. Mechanically, inhibition of BRD4 sensitized NSCLC cells to osimertinib by blocking YAP1-mediated APT1 transcription and disrupting APT1-mediated depalmitoylation of MST1 and YAP1 nuclear translocation. Collectively, inhibition of BRD4 restores osimertinib sensitivity through the APT1-MST1-YAP1 axis in NSCLC. Our study provides a novel mechanism of osimertinib resistance and suggests potential therapeutic strategies for NSCLC.

## Results

### YAP1 was upregulated, and its knockdown recovered osimertinib sensitivity

To explore the mechanisms underlying osimertinib resistance of NSCLC cells, HCC827/OR and PC-9/OR, two osimertinib-resistant cell lines, were established from parental osimertinib-sensitive HCC827 and PC-9 cells with EGFR mutation through the dose escalation method [23] (Supplementary Fig. 1A). EGFR exon 19 deletion (EGFR 19del), a major subtype of EGFR mutation with sensitivity to tyrosine kinase inhibitors (TKIs) [24]. The osimertinib-resistant HCC827/OR and PC-9/OR cell lines exhibit exon 19 deletion without EGFR C797S mutation or MET amplification. Morphologically, HCC827/OR and PC-9/OR cells appeared larger and more fibroblast-like than their parental counterparts (Supplementary Fig. 1B). Both resistant lines exhibited markedly higher IC₅₀ values for osimertinib and enhanced viability, proliferation, migration, and invasion compared with parental cells (Supplementary Fig. 1C–G), confirming successful model establishment.

**Fig. 1:**
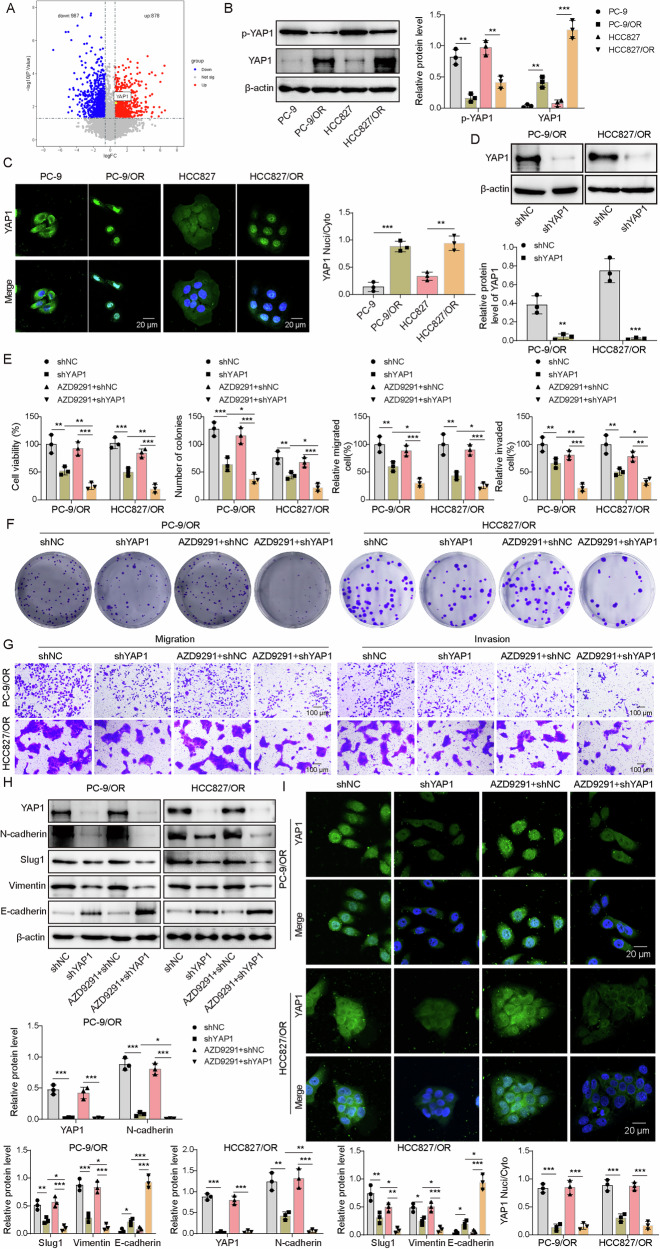
YAP1 was upregulated, and its knockdown recovered osimertinib sensitivity. **A** The expression of YAP1 in osimertinib-resistant and -sensitive NSCLC tissues was analyzed using the GEO database. **B** Western blotting analysis of p-YAP1 and active YAP1. **C**The localization of YAP1 was examined by IF staining. Scale bar, 20 μm. **D** YAP1 was knocked down, and its expression was examined by Western blotting. YAP1-knockdown PC-9/OR and HCC827/OR cells were treated with osimertinib (AZD9291) at 0.1 μM for 24 h and divided into shNC, shYAP1, AZD9291 + shNC, and AZD9291 + shYAP1 groups. **E** Cell viability was examined by CCK-8. **F** Colony formation assay was applied to assess cell proliferation. **G** Cell migration and invasion were determined with transwell assays. **H** YAP1, N-cadherin, Slug1, Vimentin, and E-cadherin were detected by Western blotting. **I** The localization of YAP1 was examined by IF staining. Scale bar, 20 μm. Mean ± SD, *n* = 3, *p < 0.05, **p < 0.01, ***p < 0.001.

Given previous reports of elevated YAP1 in osimertinib-resistant NSCLC [9], we analyzed GSE130160 data from the GEO database and found YAP1 expression significantly upregulated in resistant tissues (Fig. 1A), suggesting its potential critical role in osimertinib resistance. Consistently, resistant cells displayed higher active YAP1, evidenced by predominant nuclear localization, and reduced phosphorylated YAP1 (p-YAP1) (Fig. 1B, Supplementary Fig. 2A). Immunofluorescence analysis confirmed both the elevated nuclear accumulation of YAP1 protein in resistant cells (Fig. 1C). YAP1 depletion reduced its levels in both the cytoplasm and nucleus, whereas AZD9291 treatment alone did not alter its nuclear–cytoplasmic distribution (Fig. 1D and Supplementary Fig. 2B). Functionally, YAP1 knockdown markedly impaired cell viability, proliferation, migration, and invasion in resistant cell lines (Fig. 1E–G). Although AZD9291 alone had little effect, YAP1 knockdown sensitized resistant cells to AZD9291, resulting in a more pronounced suppression of malignant behaviors (Fig. 1E–G). Furthermore, YAP1 knockdown modulated EMT markers, reducing the expression of N-cadherin, Slug1, and Vimentin while restoring E-cadherin (Fig. 1H). AZD9291 alone did not affect YAP1 or EMT marker expression; however, in YAP1-silenced cells, AZD9291 further suppressed mesenchymal markers and enhanced E-cadherin levels (Fig. 1H). In both PC-9/OR and HCC827/OR cells, AZD9291 alone did not alter cytoplasmic or nuclear YAP1 levels, whereas YAP1 knockdown markedly reduced YAP1 in both compartments (Fig. 1I). Collectively, these results indicate that YAP1 promoted osimertinib resistance in NSCLC cells and that its inhibition restored drug sensitivity and suppressed malignant phenotypes.

To further validate the role of YAP1 in osimertinib resistance, we examined the effects of YAP1 overexpression in sensitive NSCLC cells. YAP1 was overexpressed in PC-9 and HCC827 cells, which were treated with osimertinib. Cell viability, proliferation, migration, and invasion of YAP1-overexpressing cells were increased compared with AZD9291+oe-NC or oe-NC group (Supplementary Fig. 3A–C). Furthermore, YAP1 overexpression (AZD9291+oe-YAP1 group) partially restored the inhibitory effects of AZD9291 (AZD9291+oe-NC group) on PC-9 and HCC827 cell viability, proliferation, migration, and invasion (Supplementary Fig. 3A–C). Overexpression of YAP1 upregulated N-cadherin, Slug1 and Vimentin but downregulated E-cadherin (Supplementary Fig. 3D). AZD9291 did not affect YAP1 expression but reduced N-cadherin, Slug1, Vimentin and increased E-cadherin expression, which was attenuated by YAP1 overexpression (Supplementary Fig. 3D). YAP1 overexpression increased the nuclear/ cytoplasmic YAP1 levels, while AZD9291 had no significant effect on YAP1 subcellular distribution (Supplementary Fig. 3E). Collectively, YAP1 enhanced osimertinib resistance in NSCLC cells.

### The BRD4 inhibitor reversed YAP1-mediated osimertinib resistance in NSCLC cells

Given reports implicating BRD4 in osimertinib resistance, we examined BRD4 expression in parental (PC-9 and HCC827) and resistant (PC-9/OR and HCC827/OR) cells. Resistant lines exhibited markedly higher BRD4 levels than parental counterparts (Supplementary Fig. 4A). Co-IP assays further showed that showed that BRD2 and BRD3 did not interact with YAP1, indicating specificity for BRD4 (Supplementary Fig. 4B). Dose–response experiments with the BRD4 inhibitor NHWD870 revealed no impact on cell viability at 0.1 nM, but significant reductions at concentrations ≥0.5 nM (Supplementary Fig. 4C). NHWD870 treatment reduced BRD4, active YAP1, and EMT markers (N-cadherin, Slug1, Vimentin), while increasing p-YAP1 and E-cadherin in a concentration-dependent manner (Supplementary Fig. 5A), suggesting that BRD4 may regulate YAP1-mediated EMT and drug resistance.

To confirm these pharmacological findings, BRD4 was silenced by shRNA (Supplementary Fig. 5B). Knockdown reduced cell viability and mesenchymal marker expression, while increasing p-YAP1 and E-cadherin (Supplementary Fig. 5C–D), further supporting BRD4’s functional role in YAP1-associated resistance.

**Fig. 2:**
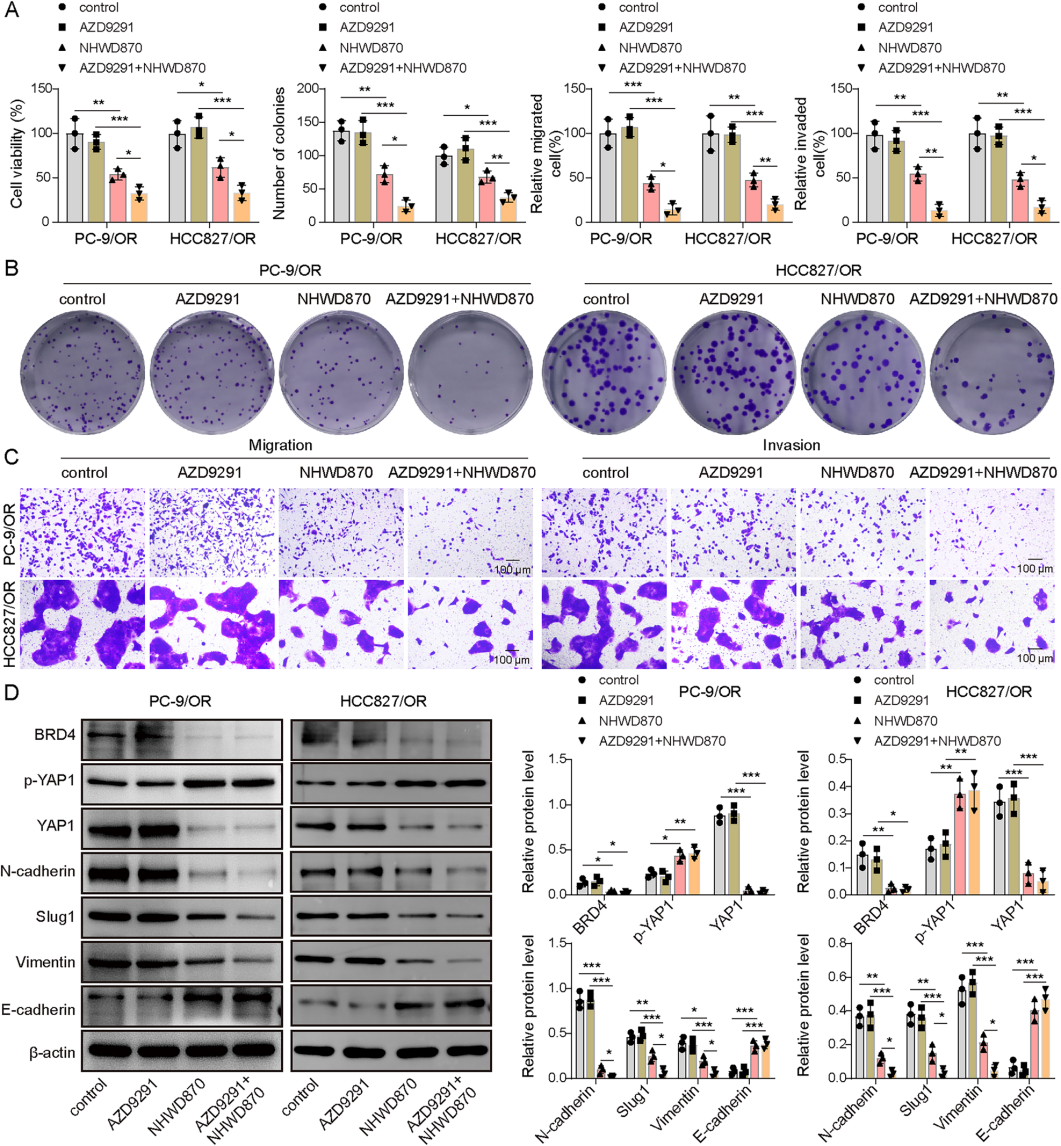
The BRD4 inhibitor reversed YAP1-mediated osimertinib resistance. PC-9/OR and HCC827/OR cells were treated with AZD9291 in combination with 5 nM NHWD870 and divided into control, AZD9291, and AZD9291 + NHWD870 (5 nM) groups. **A** Cell viability was examined by CCK-8. **B** A colony formation assay was applied to assess cell proliferation. **C** Cell migration and invasion, Scale bar, 100 μm. **D** Western blot analysis of BRD4, p-YAP1, active YAP1, N-cadherin, Slug1, Vimentin, and E-cadherin. Mean ± SD, *n* = 3, *p < 0.05, **p < 0.01, ***p < 0.001.

We next tested whether targeting BRD4 could restore osimertinib sensitivity. Resistant cells were treated with AZD9291, NHWD870, or both. AZD9291 alone did not significantly affect viability, proliferation, migration, invasion, or expression of BRD4, p-YAP1, active YAP1, or EMT markers (Fig. 2A–D). In contrast, NHWD870 significantly suppressed all these malignant phenotypes and downregulated BRD4, active YAP1, and EMT-associated molecular (N-cadherin, Slug1, Vimentin), but upregulated p-YAP1 and E-cadherin (Fig. 2A–D). These findings suggest that BRD4 inhibition alone can overcome YAP1-driven resistance in osimertinib-resistant NSCLC cells.

To assess whether these effects extend to parental cells, we treated PC-9 and HCC827 with AZD9291, NHWD870, or both. Treatment with either AZD9291 or NHWD870 alone significantly reduced cell viability and proliferation, inhibited migration and invasion, induced G1/S phase arrest, and increased apoptosis (Supplementary Fig. 6A–E). While AZD9291 did not alter BRD4 expression, it decreased active YAP1 and mesenchymal markers (N-cadherin, Slug1, Vimentin) and upregulated epithelial markers such as E-cadherin and p-YAP1 (Supplementary Fig. 6F). NHWD870 produced similar changes and also reduced BRD4. Combination treatment synergistically enhanced these molecular and phenotypic effects (Supplementary Fig. 6F). To further validate these findings, we examined the dose-dependent effects of NHWD870 in combination with AZD9291 and found that NHWD870 amplified AZD9291-mediated suppression of viability, proliferation, migration, and invasion (Supplementary Fig. 7A–C). Besides, NHWD870 potentiated the AZD9291-induced increase in p-YAP1 and E-cadherin and reduction in active YAP1 and EMT markers (Supplementary Fig. 7D). In YAP1-overexpressing cells, which exhibited enhanced malignant behaviors and EMT marker expression, NHWD870 reversed these effects, including the suppression of N-cadherin, Slug1, and Vimentin and upregulation of p-YAP1 and E-cadherin, through downregulating BRD4 levels (Supplementary Fig. 8A–D and Supplementary Fig. 9A–D). These data collectively underscore the therapeutic utility of BRD4 inhibition in overcoming YAP1-driven malignant phenotypes in both resistant and sensitive NSCLC cells.

These findings suggested that the BRD4 inhibitor NHWD870 significantly reversed YAP1-mediated osimertinib resistance.

### MST1 was significantly depalmitoylated at C699 in osimertinib-resistant cells

**Fig. 3:**
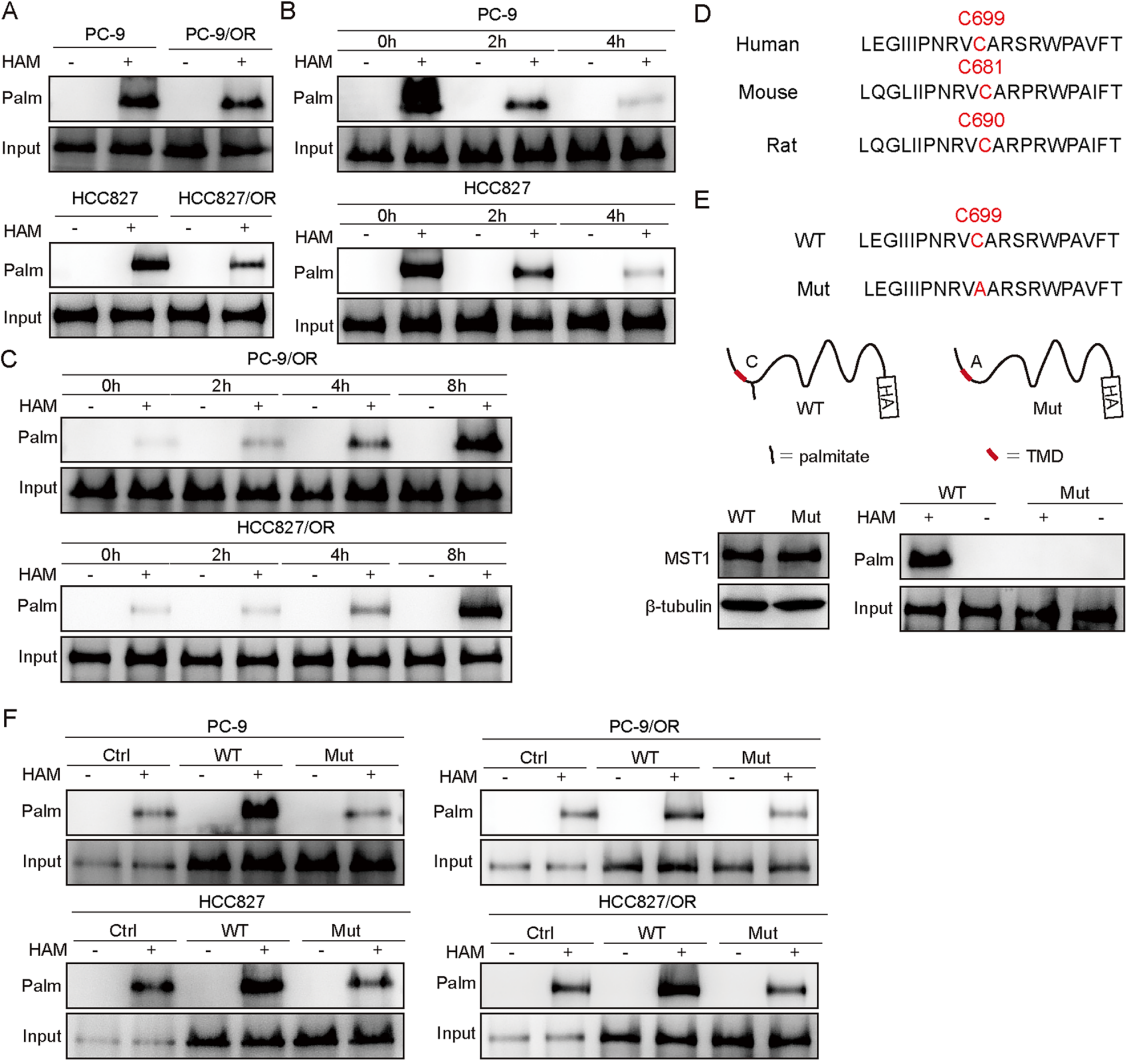
MST1 was significantly depalmitoylated at C699. **A** MST1 palmitoylation was determined through Acyl-RAC. **B** PC-9 and HCC827 cells were treated with 2-BP for 0, 2 or 4 h in the presence of hydroxylamine hydrochloride (HAM). MST1 palmitoylation was determined through Acyl-RAC in 2-BP-treated cells. **C** PC-9/OR and HCC827/OR cells were treated with Palm B for 0, 2, 4, and 8 h. MST1 palmitoylation was determined through Acyl-RAC in 2-BP-treated cells. **D** Cysteine conservation analysis of MST1 in various species (human, mouse, and rat). **E** HEK293T cells were transfected with HA-tagged wild-type or C699A mutant MST1, and MST1 palmitoylation was determined through Acyl-RAC assay. **F** Cells were transfected with HA-tagged wild-type or C699A mutant MST1, and MST1 palmitoylation was determined through Acyl-RAC assay. *n* = 3.

Given that MST1 is a key upstream regulator of YAP1 nuclear translocation, we next examined whether MST1 undergoes palmitoylation. Compared to resistant cells, osimertinib-sensitive cells exhibited higher MST1 palmitoylation (Fig. 3A). Treatment of PC-9 and HCC827 cells with 2-BP resulted in a time-dependent decrease in MST1 palmitoylation (Fig. 3B). Conversely, treatment of PC-9/OR and HCC827/OR cells with Palm B, an APT1 inhibitor, increased MST1 palmitoylation(Fig. 3C). Mass spectrometry confirmed the palmitoylation site in the MST1 peptide segment THNCWVLEGIIIPNRVC699AR (Supplementary Fig. 10A). Cysteine conservation analysis revealed that the palmitoylation site at human MST1 C699 was evolutionarily conserved (mouse C681, rat C691) (Fig. 3D). Overexpression of HA-tagged MST1 WT or C699A mutant in HEK293T cells demonstrated that palmitoylation was abolished in the C699A mutant (Fig. 3E). In NSCLC cells, WT-MST1 exhibited higher palmitoylation, whereas resistant cells showed reduced levels (Fig. 3F). Thus, osimertinib-resistant cells showed decreased palmitoylation of MST1.

Co-IP assays were used to assess other post-translational modifications of MST1. MST1 immunoprecipitates from PC-9 and PC-9/OR cells were probed for phosphorylation, acetylation, and lactylation. MST1 was phosphorylated but showed no detectable acetylation or lactylation, and phosphorylation was diminished in resistant cells (Supplementary Fig. 10B). These results indicate that MST1 was palmitoylated at C699 and that this modification was reduced in osimertinib-resistant NSCLC cells.

### Inhibiting APT1-mediated MST1 depalmitoylation recovered osimertinib sensitivity

**Fig. 4:**
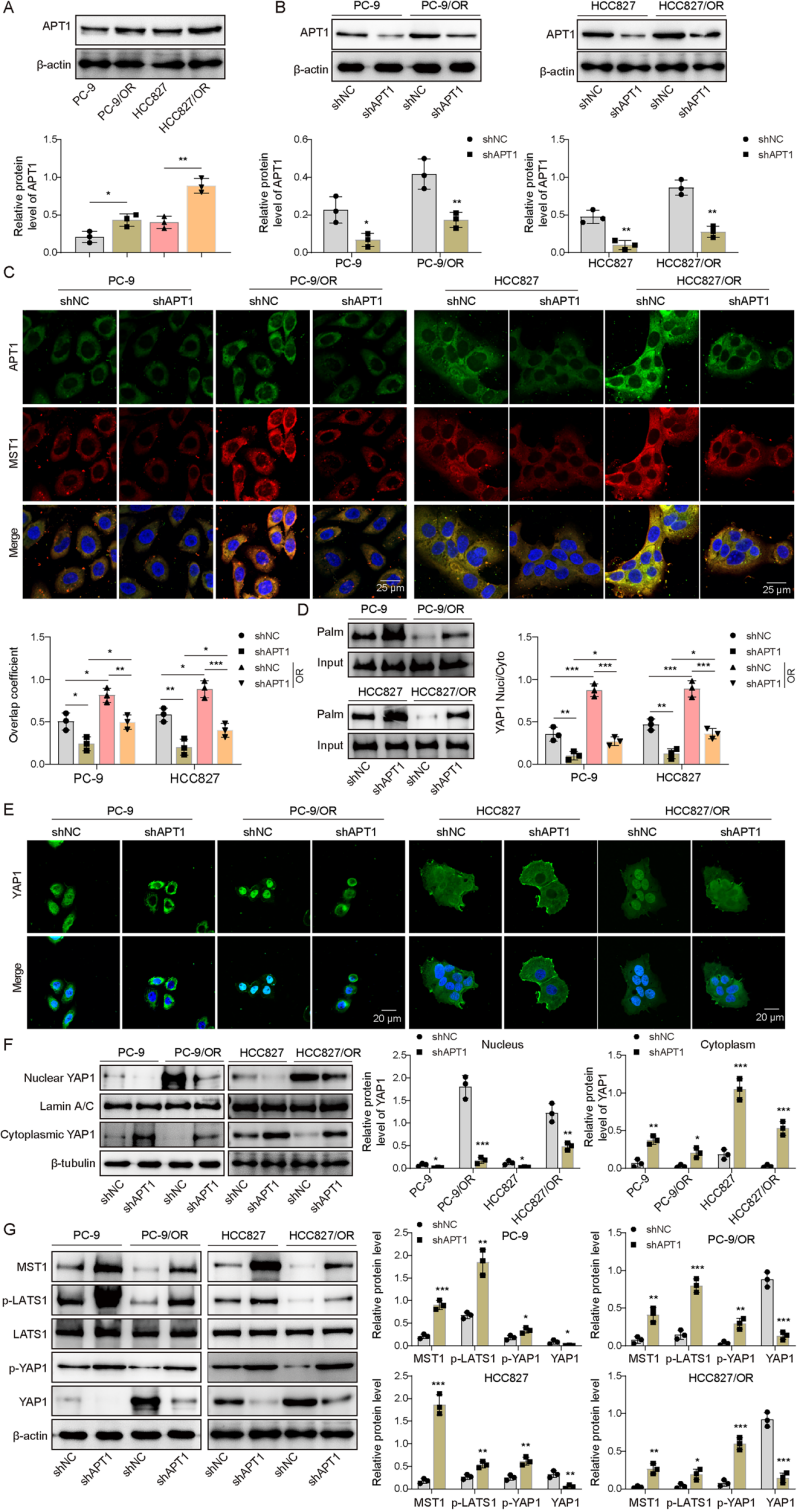
Inhibiting APT1-mediated MST1 depalmitoylation recovered osimertinib sensitivity. **A** APT1 was detected by Western blotting. APT1 was knocked down through shAPT1 transfection, and cells were divided into shNC and shAPT1 groups. **B** APT1 was detected by Western blotting. **C** The colocalization of APT1 and MST1 was assessed by IF staining. Scale bar, 20 μm. **D** MST1 palmitoylation was determined through the Acyl-RAC assay. **E** The localization of YAP1 was examined by IF staining. Scale bar, 20 μm. **F** The abundance of YAP1 in the cytoplasmic and nuclear fractions was determined with Western blotting. **G** Western blotting analysis of MST1, p-LAST1, LAST1, p-YAP1 and YAP1. Mean ± SD, *n* = 3, *p < 0.05, **p < 0.01, ***p < 0.001.

To identify specific depalmitoylases, HEK293T cells were transfected with Flag-tagged APT1, APT2, PPT1, PPT2, ABHD17A, or HA-tagged MST1. Among these, overexpression of APT1 and APT2 reduced MST1 palmitoylation, with APT1 showing the most pronounced effect (Supplementary Fig. 10C). Consequently, APT1 was selected for further investigation. Since APT1 is a primary depalmitoylase, we examined its expression and found it elevated in PC-9/OR and HCC827/OR compared to parental cells (Fig. 4A). Knockdown of APT1 via shRNA (Fig. 4B) or pharmacological inhibition with NHWD870 increased MST1 phosphorylation, with the combination producing the strongest effect (Supplementary Fig. 10D), suggesting that enhanced MST1 palmitoylation promoted phosphorylation. IF staining showed decreased co-localization of APT1 and MST1 in the cytoplasm (Fig. 4C), and Co-IP confirmed their interaction (Supplementary Fig. 10E). In resistant cells, APT1 knockdown increased MST1 palmitoylation, whereas in sensitive cells, knockdown had minimal effect (Fig. 4D). Moreover, a significant increase in YAP1 nuclear translocation was observed in osimertinib-resistant cells (Fig. 4E, F). However, knockdown of APT1 reduced the nuclear translocation of YAP1 (Fig. 4E, F). Besides, knockdown of APT1 promoted the expression of MST1, p-LATS1, and p-YAP1 but reduced active YAP1 expression in all cells, although osimertinib-resistant cells contained lower levels of these proteins (MST1, p-LATS1, and p-YAP1) than osimertinib-sensitive cells (Fig. 4G). Subsequently, APT1-knockdown PC-9/OR and HCC827/OR cells were treated with AZD9291. Knockdown of APT1 reduced cell viability, proliferation, migration, and invasion (Supplementary Fig. 11A–C). AZD9291 showed no significant effects, whereas knockdown of APT1 sensitized HCC827/OR and PC-9/OR cells to AZD9291, which was characterized by further decreased viability, proliferation, migration, and invasion (Supplementary Fig. 11A-C). Furthermore, the knockdown of APT1 downregulated APT1, active YAP1, N-cadherin, Slug1, and Vimentin but upregulated p-YAP1 and E-cadherin (Supplementary Fig. 11D). AZD9291 had no effect on the expression of these factors, but AZD9291 could reduce N-cadherin, Slug1, and Vimentin but enhance E-cadherin expression in APT1-knockdown cells, suggesting that knockdown of APT1 sensitized HCC827/OR and PC-9/OR cells to osimertinib (Supplementary Fig. 11D). Thus, APT1 enhanced osimertinib resistance of NSCLC cells by depalmitoylating MST1.

We additionally explored the EGFR–AKT signaling axis. The treatment of AZD9291 at concentrations ranging from 0 to 10^4 ^nM reduced p-EGFR/EGFR ratios in parental cells in a concentration-dependent manner but had limited effect on p-AKT/AKT levels in resistant cells, indicating persistent downstream activation (Supplementary Fig. 12A). HCC827/OR and PC-9/OR cells were divided into four groups: shNC, shYAP1, AZD9291 (10^2 ^nM) + shNC, and AZD9291 + shYAP1, and YAP1 knockdown downregulated p-AKT/AKT (Supplementary Fig. 12B). AZD9291 treatment showed limited effects on p-AKT/AKT (Supplementary Fig. 12B). HCC827/OR and PC-9/OR cells were grouped as control, AZD9291, NHWD870, and AZD9291 + NHWD870, and NHWD870 alone suppressed p-AKT/AKT ratio (Supplementary Fig. 12C). AZD9291 alone minimally impacted p-AKT/AKT (Supplementary Fig. 12C). HCC827/OR and PC-9/OR cells were divided into shNC, shAPT1, AZD9291 + shNC, and AZD9291 + shAPT1 groups, and APT1 knockdown attenuated p-AKT/AKT ratio (Supplementary Fig. 12D). AZD9291 treatment had negligible effects on p-AKT/AKT ratio (Supplementary Fig. 12D). HCC827/OR and PC-9/OR cells were divided into control, NHWD870, NHWD870 + NC, and NHWD870 + APT1. NHWD870 treatment reduced p-AKT/AKT ratio (Supplementary Fig. 12E). Subsequent APT1 overexpression reversed this suppression, further increasing p-AKT/AKT levels (Supplementary Fig. 12E). These findings indicate that APT1-mediated MST1 depalmitoylation contributed to sustained EGFR–AKT signaling, supporting a novel mechanism of drug resistance in NSCLC.

### The BRD4 inhibitor blocked YAP1-mediated APT1 transcription

**Fig. 5:**
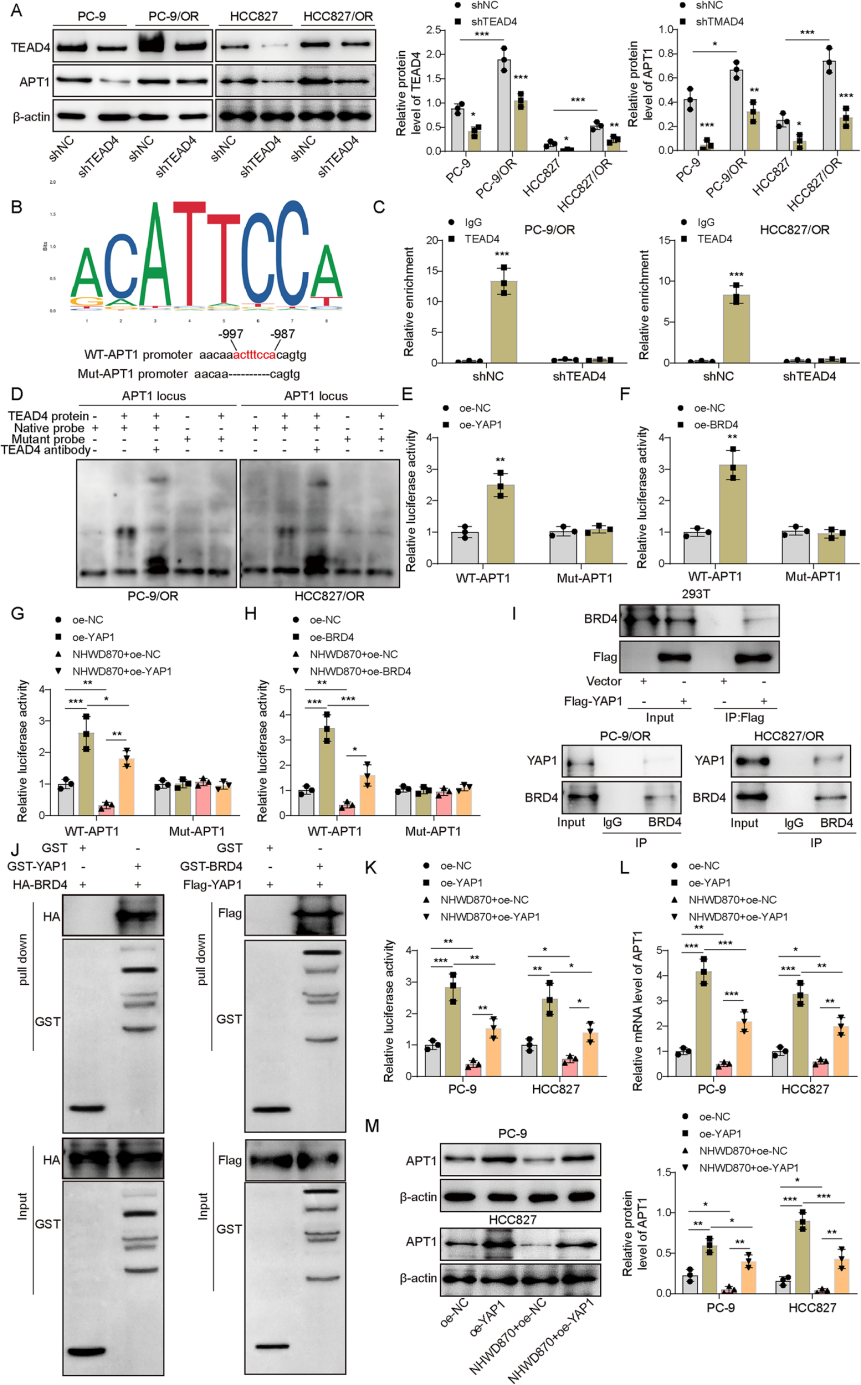
The BRD4 inhibitor blocked YAP1-mediated APT1 transcription. **A** TEAD4 was knocked down. TEAD4 and APT1 were detected by Western blotting. **B** The binding site for TEAD4 in the APT1 promoter region was predicted by the JASPAR database. **C** The enrichment of APT1 by the TEAD4 antibody was evaluated with ChIP-qPCR analysis. The PCR products were loaded for gel electrophoresis. **D** The binding of TEAD4 to the APT1 promoter was analyzed by EMSA. The native probe-TEAD4-TEAD4 antibody complex ran slowest in gel electrophoresis. **E** HEK293T cells were transfected with wild-type or mutant APT1 and Flag-tagged YAP1. YAP1-mediated recruitment of TEAD4 for binding to the APT1 promoter was assessed by luciferase activity. **F** HEK293T cells were transfected with wild-type or mutant APT1 and HA-tagged BRD4. The binding of BRD4 to the APT1 promoter was analyzed by luciferase activity. **G** HEK293T cells were transfected with wild-type or mutant APT1 and Flag-tagged YAP1 and treated with NHWD870. The binding of YAP1 to the APT1 promoter was analyzed by luciferase activity. **H** HEK293T cells were transfected with wild-type or mutant APT1 and HA-tagged BRD4 and treated with NHWD870. The binding of BRD4 to the APT1 promoter was analyzed by luciferase activity. **I** HEK293T cells were transfected with Flag-tagged YAP1, and BRD4 was co-immunoprecipitated by the Flag antibody. YAP1 was co-immunoprecipitated by the BRD4 antibody. PC-9/OR, or HCC827/OR cells were used to detect the interaction between endogenous BRD4 and YAP. **J** GST-tagged YAP1 (or BRD4) and HA-tagged BRD4 (Flag-tagged YAP1) were transfected into HEK293T, and the interaction of YAP1 and BRD4 was analyzed by GST pull-down assays. **K** YAP1-overexpressing PC-9 and HCC827 cells were treated with NHWD870 and divided into oe-NC, oe-YAP1, NHWD870+oe-NC, and NHWD870+oe-YAP1 groups, and the activity of the APT1 promoter was evaluated by luciferase activity. **L**, **M** ATP1 was detected by RT-qPCR and Western blot. Mean ± SD, *n* = 3, *p < 0.05, **p < 0.01, ***p < 0.001.

As YAP1 exerts its transcriptional function through interaction with TEAD transcription factors [25], we investigated the role of TEAD4 in APT1 regulation. Knockdown of TEAD4 via shTEAD4 transfection significantly reduced APT1 expression (Fig. 5A). JASPAR database analysis predicted a TEAD4-binding site in the APT1 promoter (–997 to –987) (Fig. 5B). ChIP assays confirmed that APT1 was effectively enriched by the TEAD4 antibody, which was abrogated by TEAD4 knockdown (Fig. 5C). EMSA assays showed that the TEAD4 antibody-TEAD4-native probe complex ran slowest in the electrophoresis, followed by the TEAD4-native probe complex, suggesting that TEAD4 bound to the APT1 promoter (Fig. 5D). Overexpression of YAP1 or BRD4 increased wild-type APT1 promoter luciferase activity but not mutant promoter activity (Fig. 5E, F), implying that YAP1 and BRD4 might recruit TEAD4 to the APT1 promoter. Overexpression of YAP1 or BRD4-mediated increased luciferase activity was reduced by NHWD870 treatment, but neither overexpression of YAP1 or BRD4 nor NHWD870 treatment influenced luciferase activity of mutant APT1 promoter (Fig. 5G, H). Co-IP assays exhibited that BRD4 could be co-immunoprecipitated by the antibody against Flag-tagged YAP1, and YAP1 was co-immunoprecipitated by the BRD4 antibody (Fig. 5I). GST-pulldown assays further confirmed the interaction between YAP1 and BRD4 (Fig. 5J). YAP1 was overexpressed in PC-9 and HCC827 cells, and the luciferase activity of the wild-type APT1 reporter was greatly increased by YAP1 overexpression and decreased by NHWD870 treatment, which reversed the effect of YAP1 overexpression (Fig. 5K). APT1 was upregulated by YAP1 overexpression, but it was reversed by NHWD870 treatment (Fig. 5L, M). Thus, inhibition of BRD4 suppressed YAP1-mediated APT1 transcription.

### The BRD4 inhibitor disrupted APT1-mediated depalmitoylation of MST1 to restore osimertinib sensitivity

**Fig. 6:**
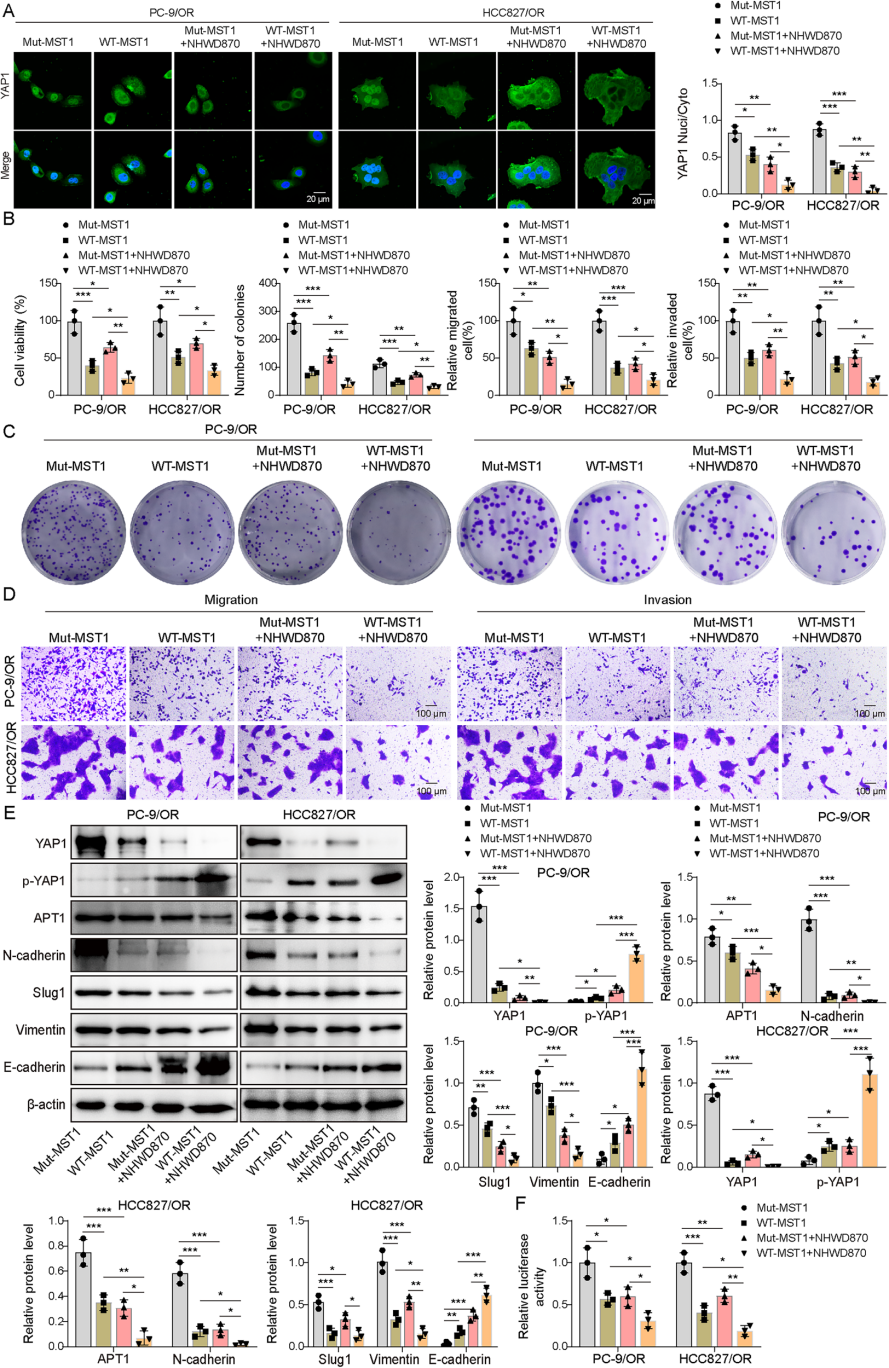
The BRD4 inhibitor disrupted APT1-mediated depalmitoylation of MST1 to restore osimertinib sensitivity in osimertinib-resistant NSCLC cells. PC-9/OR and HCC827/OR cells were transfected with C699 wild-type or mutant HA-tagged MST1 and treated with NHWD870, and cells were divided into WT-MST1, Mut-MST1, WT-MST1 + NHWD870, and Mut-MST1 + NHWD870 groups. **A** The localization of YAP1 was examined by IF staining. Scale bar, 20 μm. **B** Cell viability. **C** Colony formation assay. **D** Cell migration and invasion, Scale bar = 100 μm. **E** Western blotting analysis of p-YAP1, YAP1, N-cadherin, Slug1, Vimentin, and E-cadherin. **F** The activity of the APT1 promoter was evaluated by luciferase activity. Mean ± SD, *n* = 3, *p < 0.05, **p < 0.01, ***p < 0.001.

To investigate the role of APT1 in regulating MST1 palmitoylation and osimertinib sensitivity, PC-9/OR and HCC827/OR cells were transfected with HA-tagged WT or C699 Mut MST1 and treated with NHWD870. Compared to the Mut-MST1 group, nuclear/cytoplastic YAP1 was decreased in the WT-MST1 and NHWD870 + Mut-MST1 group, and YAP1 was further reduced the nuclear/cytoplastic YAP1 in the NHWD870 + WT-MST1 group (Fig. 6A). Cell viability, proliferation, migration, and invasion were inhibited in the WT-MST1 and NHWD870 + Mut-MST1 group and further reduced in the NHWD870 + WT-MST1 group (Fig. 6B–D). Additionally, the levels of p-YAP1 and E-cadherin were increased but the expression of active YAP1, APT1, N-cadherin, Slug1 and Vimentin was inhibited in the WT-MST1 and NHWD870 + Mut-MST1 group, and these effects on their expression were further strengthened in the NHWD870 + WT-MST1 group (Fig. 6E). Furthermore, compared to the Mut-MST1 group, the luciferase activity of the wildtype APT1 reporter was suppressed in the WT-MST1 and NHWD870 + Mut-MST1 group, which was further reduced in the NHWD870 + WT-MST1 group (Fig. 6F). Subsequently, APT1 was overexpressed, and cells were treated with NHWD870. p-YAP1 was upregulated, APT1 was downregulated and the luciferase activity of the wildtype APT1 reporter was reduced after NHWD870 treatment, but APT1 overexpression reversed these effects (Supplementary Fig. 13A, B). Moreover, NHWD870 treatment facilitated MST1 palmitoylation and inhibited YAP1 nuclear translocation, which was abolished by APT1 overexpression (Supplementary Fig. 13C, D). Cell viability, proliferation, migration, and invasion were reduced after NHWD870 treatment, but APT1 overexpression reversed these effects (Supplementary Fig. 13E–G). In addition, PC-9 and HCC827 cells were transfected with mutant or wild-type MST1, and cell viability, proliferation, migration, and invasion were evaluated. Compared to PC-9 and HCC827 cells transfected with MST1-WT, those transfected with MST1-Mut exhibited increased cell viability, enhanced proliferation, and augmented migration and invasion capabilities (Supplementary Fig. 14A–C). Following AZD9291 treatment, both the MST1-WT and MST1-Mut group showed decreased cell viability, suppressed proliferation, and reduced migratory and invasive capacities (Supplementary Fig. 14A–C). In conclusion, inhibition of BRD4 suppressed APT1-mediated depalmitoylation of MST1 to recover osimertinib sensitivity.

### The BRD4 inhibitor enhanced osimertinib sensitivity of NSCLC cells in mice

**Fig. 7:**
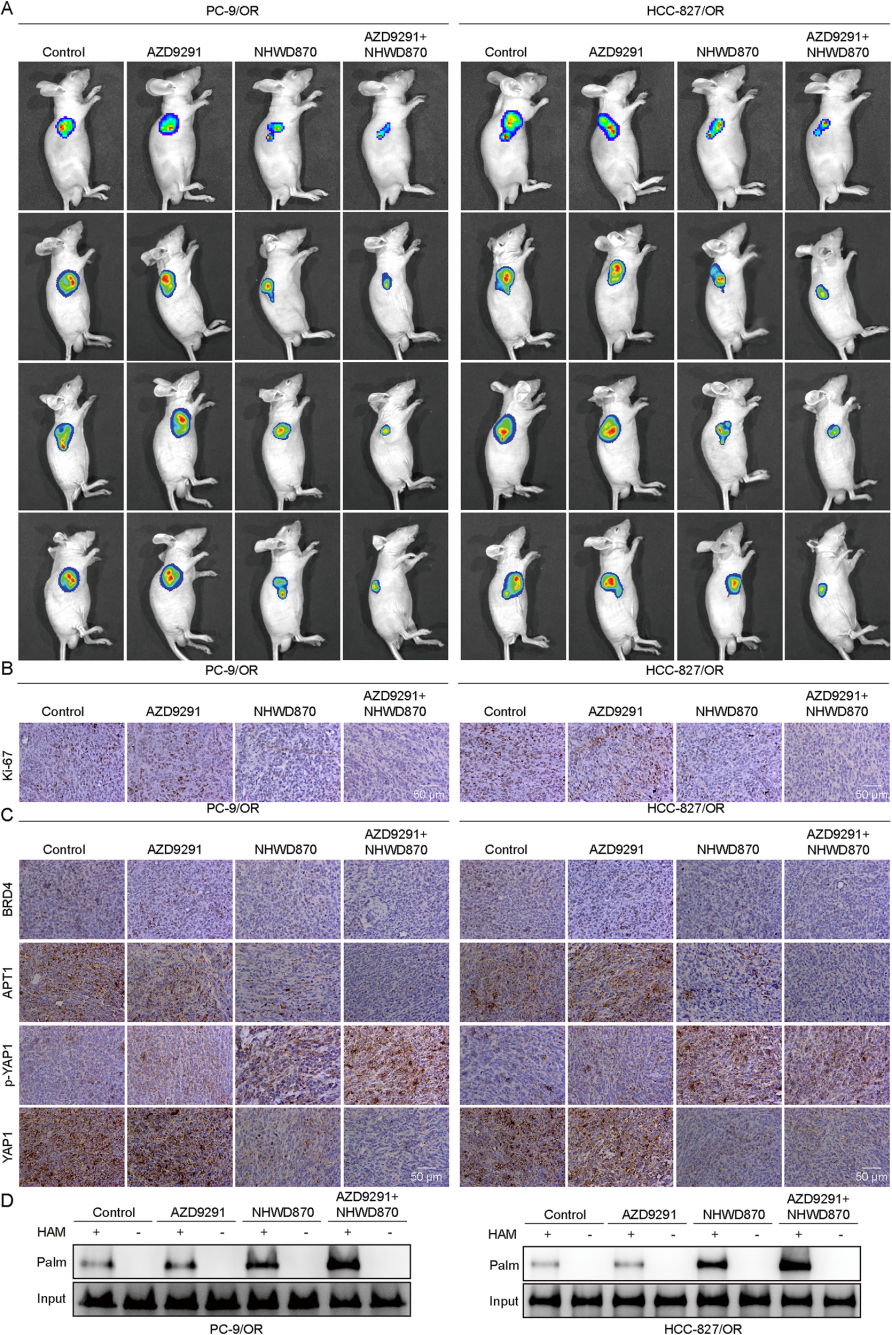
The BRD4 inhibitor enhanced osimertinib sensitivity of NSCLC cells in mice. PC-9/OR and HCC827/OR cells were subcutaneously injected into mice, and mice were treated with AZD9291 or/and NHWD870 and divided into control, AZD9291, NHWD870, and AZD9291 + NHWD870 groups. **A** In vivo imaging to observe the size of the tumor. **B** The proliferation of tumor cells was determined by Ki-67 staining. Scale bar, 50 μm. **C** IHC staining of BRD4, APT1, YAP1, and p-YAP1 in the tumors. Scale bar, 50 μm. **D** MST1 palmitoylation was determined through the Acyl-RAC assay. Mean ± SD, *n* = 6.

Having demonstrated that BRD4 inhibition can reverse osimertinib resistance in vitro, we next evaluated its effects in vivo. In a separate model, PC-9/OR and HCC827/OR cells were subcutaneously injected into mice, and mice were treated with AZD9291 and/or NHWD870. The results revealed that while AZD9291 alone had a minimal impact on tumor size, NHWD870 treatment significantly reduced tumor size, with the most pronounced effect observed in the combination treatment (Fig. 7A), suggesting that tumor growth was inhibited by both NHWD870 and AZD9291 treatments. Ki67, a well-known cell proliferation marker, staining showed that tumor cell proliferation was not affected by the treatment of AZD9291 alone and inhibited by the treatment of NHWD870 alone (Fig. 7B). However, mice treated with NHWD870 and AZD9291 showed the most significant reduction in cell proliferation (Fig. 7B). The treatment of AZD9291 alone did not affect BRD4, APT1, YAP1, and p-YAP1 and MST1 palmitoylation in tumor cells, whereas the treatment of NHWD870 alone reduced the expression of BRD4, APT1 and YAP1 but enhanced YAP1 phosphorylation and MST1 palmitoylation (Fig. 7C, D). The most pronounced decrease for BRD4, APT1, and p-YAP1 and increase for MST1 palmitoylation were observed in mice treated with NHWD870 in combination with AZD9291 (Fig. 7C, D).

Besides, PC-9 and HCC827 cells were injected into mice via the tail vein, followed by treatment with AZD9291 or the BRD4 inhibitor NHWD870. Mice were divided into control, AZD9291, NHWD870, and AZD9291 + NHWD870 groups. Treatment with AZD9291 or NHWD870 alone led to decreased liver lesions and a lower number of liver nodules (Supplementary Fig. 15A–C). Combination treatment further relieved liver pathology, resulting in a significant decrease in liver nodules (Supplementary Fig. 15A–C).

Collectively, the BRD4 inhibitor the sensitivity of NSCLC cells to osimertinib in vivo.

## Discussion

Lung cancer caused an estimated 1.8 million deaths in 2020 [26]. NSCLC accounts for 85–90% of lung cancer cases [27]. One of the significant oncogenic drivers is the EGFR mutations, including classical activating mutations (Exon 19 deletion and L858R mutation), which account for approximately 90% of EGFR mutations [28]. EGFR-TKIs, including osimertinib, offer great clinical significance for patients carrying classical EGFR activating mutations [29]. However, the greatest challenge for the clinical application of osimertinib is that almost all patients inevitably develop osimertinib resistance. Emerging evidence has revealed novel mechanisms underlying osimertinib resistance, such as tertiary EGFR mutations, Kirsten rat sarcoma viral oncogene homologue (KRAS) mutations, phosphoinositide 3-kinase (PI3K) pathway activation, and oncogenic fusions [30]. Exploring mechanisms of osimertinib resistance contributes to developing novel approaches to counteract resistance to osimertinib. Here, we found that inhibition of BRD4 enhanced osimertinib sensitivity by suppressing YAP1-mediated APT1 transcription and subsequently disrupting MST1 depalmitoylation and the nuclear translocation of YAP1 in NSCLC, revealing a novel mechanism underlying osimertinib resistance.

YAP1 plays vital roles in the resistance to multidrug in various cancers. Upregulation of nuclear YAP1 is associated with poor survival, and YAP1 enhances the resistance to drugs through CD74 signaling in small cell lung cancer [31, 32]. Overexpression of YAP1 promotes the development of resistance to enzalutamide by enhancing lipid metabolism and cancer stemness in prostate cancer [33]. Intriguingly, recent studies have demonstrated YAP1 as a novel key player in the acquired osimertinib resistance in NSCLC. Especially, the nuclear translocation of YAP1 is a key process for osimertinib resistance in NSCLC [9, 10, 34]. In this study, we confirmed increased nuclear YAP1 and decreased phosphorylated YAP1 in osimertinib-resistant NSCLC cells. Silencing of YAP1 significantly counteracted osimertinib resistance, whereas overexpression of YAP1 greatly inhibited osimertinib sensitivity, supporting the notion that YAP1 promotes drug resistance in NSCLC. BRD4 regulates the Hippo/YAP signaling [35], and inhibition of BRD4 with the BET inhibitor NHWD870 represses YAP1 expression via blocking the binding of BRD4 to the YAP1 promoter in melanoma [36]. Consistently, we found that BRD4 was highly expressed, and NHWD870-mediated inhibition of BRD4 enhanced YAP1 phosphorylation and significantly reversed YAP1-mediated osimertinib resistance. Our findings reveal novel roles of BRD4 and YAP1 in osimertinib resistance.

The complex of MST1/2-WW domain containing protein 1 (WW45) phosphorylates and activates the LATS1/2-Mps one binder 1 A and B (MOB1A/B) complex that in turn phosphorylates YAP/TAZ and blocks nuclear localization [37]. Increased MST1 expression may cause apoptosis by depleting YAP1 in high glucose-exposed cardiomyocytes [38]. MST1 is downregulated in osimertinib-resistant NSCLC cells, and blocking MST1 may promote the nuclear translocation of YAP1 in EGFR TKI-resistant NSCLC cells [9]. These studies suggest the implication of MST1 in osimertinib resistance by regulating YAP1. Protein palmitoylation is implicated in drug resistance [39, 40], but it has never been reported in osimertinib resistance. We first reported a novel palmitoylation site of MST1 at C699 in NSCLC cells, and osimertinib-resistant NSCLC cells exhibited increased depalmitoylation, implying the involvement of MST1 depalmitoylation in osimertinib resistance. APT1 functions as an important depalmitoylating enzyme to exert its activity in various physiological and pathological processes [41, 42]. We observed increased APT1 in osimertinib-resistant cells, and further exploration revealed that APT1 could promote osimertinib resistance by depalmitoylating MST1 and enhancing YAP1 nuclear translocation, revealing a novel mechanism by which APT1 regulates osimertinib resistance in NSCLC through depalmitoylation.

BRD4 can recruit chromatin and transcription modulators to regulate gene expression by binding to acetylated histone, non-histone proteins, and active promoters and enhancers [43, 44]. Inhibitors targeting BRD4 show great potential for cancer therapy [45]. However, the linkage between BRD4 and APT1 has never been established. Intriguingly, we found that inhibition of BRD4 with the treatment of NHWD870 could repress APT1 transcription mediated by the nuclear translocation of YAP1 in NSCLC cells for the first time. Furthermore, NHWD870 treatment restored osimertinib sensitivity by disrupting APT1-mediated depalmitoylation of MST1, indicating that targeting BRD4 may contribute to treating osimertinib-resistant NSCLC.

In conclusion, we demonstrate that inhibition of BRD4 suppresses APT1 expression and subsequently promotes MST1 palmitoylation and blocks the nuclear translocation of YAP1, thereby sensitizing NSCLC cells to osimertinib. Our study provides a novel mechanism underlying osimertinib resistance and potential therapeutic targets and approaches for counteracting osimertinib resistance in NSCLC. However, to achieve clinical application, some limitations need to be solved in future studies, such as further exploration of the molecular mechanism of osimertinib resistance, more involvement of clinical specimens and animal assays, and screening of other inhibitors that target the APT1-MST1-YAP1 axis.

## Methods

### Cell culture and treatment

PC-9 (RIKEN Cell Bank, Ibaraki, Japan), HCC827 (ATCC, VA, USA), and H1975 (ATCC) harboring a deletion in exon-19, were kept in RPMI1640 medium containing 10% (v/v) fetal bovine serum (FBS), 50 g/mL streptomycin, and 100 U/mL penicillin (all from Gibco, Carlsbad, USA) at 37 °C supplied with 5% CO_2_. To establish osimertinib-resistant PC-9/OR, HCC827/OR cells, and H1975/OR, parental PC-9, HCC827, and H1975 cells were treated with stepwise escalation of concentrations of osimertinib (AZD9291, Selleck, Houston, TX, USA) at 5 nM to 2 μM for 6 months [46]. All cells were authenticated by STR profiling and tested for mycoplasma contamination. Cells were treated with AZD9291 at 0, 0.001, 0.01, 0.1, 1, 2.5 or 5 μM and/or NHWD870 (a BET inhibitor, Selleck) at 0, 0.1, 0.5, 1, 5 or 10 nM for 8 h, or the palmitoylation inhibitor 2-bromopalmitate acid (2-BP, Selleck) at 100 μM for 0, 2 or 4 h.

### Cell transfection

Short hairpin RNAs (shRNAs) against YAP1, BRD4, APT1, and TEAD4 and scramble negative controls (shNC, GenePharma, Shanghai, China) were inserted into pLKO.1 vector (NovoPro, Shanghai, China) as gene knockdown constructs. Coding sequences for YAP1, Flag-tagged YAP1, GST-tagged YAP1, HA-tagged wild-type and mutant (C699A) MST1, HA-tagged BRD4, and Flag-tagged depalmitoylases (APT1, APT2, PPT1, PPT2, ABHD17A) were cloned into the pcDNA3.1 vector (NovoPro) for gene overexpression. The MST1 mutant was generated by site-directed mutagenesis using the QuikChange II XL Site-Directed Mutagenesis Kit (Agilent Technologies, Cat. No. 200521) according to the manufacturer’s instructions. The empty pcDNA3.1 vector served as a negative control. NSCLC cells and HEK293T cells were transfected with these constructs using Lipofectamine 3000 (Thermo Fisher Scientific, Waltham, MA, USA) for 48 h.

### Cell counting Kit-8 (CCK-8) assay

Briefly, the cell medium was removed and replaced with 100 μL fresh medium after the indicated treatment. CCK-8 (10 μL, Beyotime, Shanghai, China) was added, and cells were further incubated for 1 h. The absorbance was measured at 450 nm.

### Colony formation

Colony formation was performed to evaluate cell proliferative capacity. Cells were detached and washed, and then pelleted after centrifugation. Then, cells were resuspended, seeded into six-well plates (~ 1 × 10^3^ cells each well), and cultured for a week. Cell colonies were washed, stained with crystal violet (Beyotime), and counted.

### Transwell assay

NSCLC cell migrative and invasive capacities were evaluated by transwell assays. To analyze cell migration, after indicated treatment, NSCLC cells (~ 1 × 10^5^) were rinsed and seeded into the upper chamber of transwell chambers (8 µm pore size), and fresh complete DMEM/10% FBS medium was added into the lower chamber. The chamber was incubated for 12 h. For cell invasion examination, a similar procedure was applied except that Matrigel (YEASEN, Shanghai, China) was added to pre-coat the upper chamber, and the chamber was incubated for 24 h. Then, cells that migrated or invaded into the lower chamber were stained with crystal violet and imaged.

### MST1 palmitoylation analysis

The palmitoylation of MST1 was examined through the acyl resin-assisted capture (Acyl-RAC) method as previously described [47]. In brief, cell lysates were prepared, and free thiol groups were blocked. Subsequently, the cysteine-acyl thioester bond with neutral hydroxylamine was selectively cleaved to expose cysteine thiol groups. The lapidated cysteines were captured using a thiol-reactive resin. The S-acylated proteins were eluted and enriched for detection through Western blot.

### Western blot

The Protein Extraction Kit was purchased from Abcam (Cambridge, UK) and used to extract total protein from NSCLC cells after the indicated treatment. Protein concentration was determined with the BCA protein quantification kit (Beyotime). Protein was loaded for electrophoresis and transferred onto PVDF membranes. Then, membranes were blocked and incubated with the primary antibody against p-YAP1 (1:1000, ab226760, Abcam), active YAP1 (1:1000, ab205270, Abcam), N-cadherin (1:5000, ab76011, Abcam), E-cadherin (1:1000, ab40772, Abcam), Vimentin (1:1000, ab8978, Abcam), Slug1 (1:1000, ab183760, Abcam), BRD2 (1:1000, ab243865, Abcam), BRD3 (1:50, ab50818, Abcam), BRD4 (1:2000, ab314433, Abcam), BRD4 (1:2000, ab314433, Abcam), MST1 (1:500, ab76822), phosphorylated-MST1 (p-MST1, 1:500, PA5-104616, Thermo Fisher Scientific), APT1 (1:1000, ab91606, Abcam), p-LATS1 (1:500, AP1517, ABclone, Wuhan, Hubei, China), LAST1/2 (1:500, A18286, ABclone), TEAD4 (1:1000, ab308621, Abcam), p-EGFR (1:1000, 44-788 G, Thermo Fisher Scientific), EGFR (1:1000, MA5-13070, Abcam), p-AKT (1:1000, 44-621 G, Thermo Fisher Scientific), AKT (1:1000, AHO1112, Thermo Fisher Scientific), β-actin (1:1000, ab8227, Abcam), Lamin B1 (1:1000, ab16048, Abcam), Lamin A/C (1:1000, ab315838, Abcam), pan lactic acid-lysine (pan-Kla, 1:1000, SAB5701141, Sigma-Aldrich), Ac-K (1:1000, MA1-2021, Thermo Fisher Scientific), and phosphoserine (1:1000, ab9332), GAPDH(1:1000, ab9485, Abcam) or β-tubulin (1:500, ab6046, Abcam). After washing, membranes were incubated with HRP-conjugated secondary antibody for 1 h. Antibodies were provided by Abcam, otherwise indicated. Bands were then visualized by adding ECL substrate (Beyotime), and their intensity was analyzed using ImageJ software. The full and uncropped western blots was provided in the file named as Supplemental Materials.

### Subcellular fractionation

Cytoplasmic and nuclear protein fractions were isolated using the Nuclear and Cytoplasmic Protein Extraction Kit (Beyotime) according to the manufacturer’s instructions. Briefly, PC-9/OR and HCC827/OR cells were collected and lysed in cytoplasmic extraction buffer, followed by centrifugation to separate the cytoplasmic supernatant. The nuclear pellet was then resuspended in nuclear extraction buffer to obtain the nuclear fraction. GAPDH and Lamin B1 were used as markers for the cytoplasmic and nuclear fractions, respectively.

### Flow cytometry

Following transfection and treatment, 1 × 10⁶ cells were collected by centrifugation at 1000 rpm for 5 min and resuspended in 1 mL of cold phosphate-buffered saline (PBS). Apoptosis was evaluated using the Annexin V-FITC Apoptosis Detection Kit (Beyotime, C1062), according to the manufacturer’s instructions. Stained cells were analyzed using a FACScan flow cytometer (Becton Dickinson, San Jose, CA, USA), and data were processed with FlowJo software (v10).

### Cell cycle analysis

Cells were seeded into 6-well plates and treated with AZD9291, NHWD870, or their combination for 48 h. After treatment, cells were harvested, washed twice with cold PBS, and fixed in 70% ethanol at 4 °C overnight. Fixed cells were washed, resuspended in PBS containing 50 µg/mL propidium iodide (PI; Sigma-Aldrich) and 100 µg/mL RNase A, and incubated for 30 min at room temperature in the dark. DNA content was analyzed using a flow cytometer (Becton Dickinson), and cell cycle phase distribution (G1, S, G2) was determined with FlowJo software (v10).

### Reverse transcription quantitative real-time PCR (RT-qPCR)

Total RNA was isolated from NSCLC cells using TRIzol reagent (Thermo Fisher Scientific). The reverse transcription was performed using High-Capacity cDNA Reverse Transcription Kit (Thermo Fisher Scientific). APT1 expression was assessed through quantitative PCR with SYBR Green qPCR Mix (Beyotime) and normalized to β-actin. The 2^−∆∆Ct^ method was used to analyze the fold change of gene expression. Primers were shown in Table 1.

**Table 1:** RT-qPCR primers.

Name	Sequences
Human APT1	Forward: 5′-CAGAAACTGGCAGGTGTCAC-3′
	Reverse: 5′-GGTCACATTGGCTGGATTCA-3′
Human β-actin	Forward: 5′-CACCATTGGCAATGAGCGGTTC-3′
	Reverse: 5′-AGGTCTTTGCGGATGTCCACGT-3′

### Immunofluorescence (IF) staining

After indicated treatment, NSCLC cells were fixed in 4% paraformaldehyde and permeabilized in 0.3% Triton X-100 solution for 15 min. Cells were rinsed, blocked in 5% normal goat serum solution for 1 h. Subsequently, cells were incubated with the antibody for YAP1 (1:500, ab205270, Abcam), APT1 (1:50, MA5-34855, Thermo Fisher Scientific) or MST1 (1:100, ab51134, Abcam) overnight. Cells were incubated with fluorescent goat anti-rabbit secondary antibody for 1 h. Cells were stained with DAPI (Beyotime), washed, and mounted for imaging.

### Chromatin immunoprecipitation (ChIP) assay

NSCLC or HEK293T cells were crosslinked in 2% formaldehyde solution for 30 min on ice and washed, and cell lysates were prepared for ultrasonication to obtain 500–1000 bp chromatin fragments. The antibody for YAP1 (5 µg, 14074, Cell Signaling Technology, Danvers, MA, USA), BRD4 (5 µg, 13440, Cell Signaling Technology), or TEAD4 (5 µg, ab308621, Abcam) was added to the fragments, and the sample was mixed and incubated overnight. Normal IgG (5 µg) was used as a negative control. Protein A/G magnetic beads (Thermo Fisher Scientific) were added to the samples and incubated for another 2 h. Beads were washed, and the chromatin complexes were precipitated. The chromatin complexes were de-crosslinked, and DNA was recovered. The enrichment of the APT1 promoter was analyzed by quantitative PCR.

### Electrophoretic mobility shift assay (EMSA)

Nuclear protein was extracted from NSCLC cells after the indicated treatment using the Nuclear Extraction Kit (Abcam) and used for EMSA. The biotin-conjugated and unconjugated APT1 promoters were synthesized by Sangon (Shanghai, China). LightShift Chemiluminescent EMSA Kit was provided by Thermo Fisher Scientific to perform the EMSA assay. The TEAD4 antibody (2 µg, ab308621) was added, and a supershift band was observed in electrophoresis.

### Co-Immunoprecipitation (Co-IP)

For exogenous Co-IP, HEK293T cells were transfected as indicated, lysed in ice-cold lysis buffer, and clarified by centrifugation. Cell lysates were incubated overnight at 4 °C with 5 µg anti-Flag (ab205606, Abcam), 5 µg anti-BRD4 (13440, Cell Signaling Technology), or 2 µg anti-HA (ab49969, Abcam). Normal IgG was used as the negative control. The following day, protein A/G magnetic beads (Thermo Fisher Scientific) were added and incubated for 2 h at 4 °C. Beads were washed, and bound proteins were eluted for Western blot analysis of BRD4, YAP1, or other indicated proteins.

For endogenous Co-IP, PC-9/OR and HCC827/OR cells were lysed and clarified by centrifugation. Dynabeads Protein G (Thermo Fisher Scientific) were pre-incubated for 4 h at 4 °C with 5 µg of anti-IgG, anti-BRD4 (13440, Cell Signaling Technology), anti-MST1 (ab232551, Abcam), or anti-YAP1 (ab52771, Abcam), and then added to the cell lysates for immunoprecipitation. After incubation, beads were washed, and co-precipitated proteins were analyzed by Western blotting.

### GST pull-down assay

GST-fused YAP1 or BRD4 and HA-fused BRD4 or Flag-fused YAP1 were overexpressed in HEK293T cells, and cells were lysed on ice and subjected to centrifugation to collect cell lysates. Glutathione Sepharose (Abcam) was mixed thoroughly, washed, and added to the cell lysates. The samples were then incubated for 10 min, and GST-fused YAP1 or BRD4 was pulled down. The protein complexes were eluted and subjected to Western blotting for detecting the abundance of HA-BRD4 or Flag-YAP1.

### Dual-luciferase reporter assay

The APT1 promoter region containing the wild-type or mutant binding sites for YAP1 and BRD4 was cloned into the pGL3-Enhancer Vector (Promega, Madison, WI, USA). HEK293T or NSCLC cells were co-transfected with the APT1 promoter reporter and Flag-YAP1, HA-BRD4, YAP1, or WT/Mut-HA-MST1. The Dual-Glo Luciferase Assay System (Promega) was used to analyze the luciferase activity after 48 to 72 h.

### A subcutaneous mouse model for NSCLC

BABL/c nude mice (aged 6 weeks and weighing between 16–20 grams) were purchased from Human SJA laboratory animal co., LTD (Hunan, China) and blindly and randomly divided into control, AZD9291, NHWD870, and AZD9291 + NHWD870 groups. PC-9/OR and HCC827/OR cells were subcutaneously injected into the right flanks (5 × 10^6^ cells per mouse). Mice in the AZD9291, NHWD870, and AZD9291 + NHWD870 groups received injections of AZD9291 (1.25 mg/kg), NHWD870 (1.5 mg/kg), and AZD9291 (0.625 mg/kg) plus NHWD870 (0.75 mg/kg) once a day. After 4 weeks, tumors were excised. Tumor growth was monitored by in vivo bioluminescence imaging (IVIS Spectrum, PerkinElmer) and subsequent immunohistochemistry staining. Animal procedures were conducted by the Institutional Animal Care guidelines of Xiangya Hospital, Central South University, and approved by the Animal Care and Use Committee of Xiangya Hospital, Central South University, No.2022020637.

In total, 24 mice were used for the experiments. PC-9 or HCC827 cells (1 × 10^6^ cells in 100 µL PBS) were injected into the lateral tail vein. One week after injection, mice were randomly assigned to four groups (*n* = 6 per group) and treated via oral gavage with vehicle control, AZD9291 (1.25 mg/kg), NHWD870 (1.5 mg/kg), or their combination (0.625 mg/kg AZD9291 plus 0.75 mg/kg NHWD870) once daily for 4 weeks. At the end of treatment, mice were euthanized, livers were harvested, photographed, and metastatic nodules were counted under a dissecting microscope. All animal experiments were approved by the Institutional Animal Care and Use Committee and performed in accordance with relevant guidelines and regulations.

### Hematoxylin and Eosin (H&E) Staining

Liver samples were fixed in 4% paraformaldehyde (Servicebio, Wuhan, Hebei, G1101) at 4 °C for 24 h, dehydrated through a graded ethanol series, cleared in xylene (Sinopharm, Beijing, China, 10023418), and embedded in paraffin. Paraffin-embedded tissues were sectioned at a thickness of 4 μm using a microtome. Sections were deparaffinized in xylene, rehydrated through descending ethanol concentrations, and rinsed in distilled water. The sections were then stained with hematoxylin solution (Beyotime, C0107) for 5 min, rinsed in running tap water, and differentiated in 1% acid alcohol. After bluing in running water for 10 min, sections were counterstained with eosin Y solution (Beyotime, C0109) for 1–2 min, followed by dehydration in ascending ethanol concentrations and clearing in xylene. Stained sections were mounted with a coverslip using neutral resin (Solarbio, Beijing, China, G8590) and visualized under a light microscope.

### Immunohistochemistry (IHC) staining

Tumor tissues were embedded in paraffin, and sections at 5 µm were prepared. Sections were deparaffinized, rehydrated, and subjected to antigen retrieval. After block, sections were incubated with the antibody for p-YAP1 (1:50, ab226760, Abcam), YAP1 (1:100, ab205270, Abcam) and Ki-67 (1:200, ab15580, Abcam). Sections were rinsed and incubated with the HRP-labelled secondary antibody for 1 h. The signal was visualized by adding DAB (Beyotime) and detected after hematoxylin (Beyotime) counterstaining.

### GEO dataset analysis

Gene expression data were obtained from the GEO database (accession number GSE130160), containing 12 samples: 6 patient-derived xenograft tumors (2 osimertinib-sensitive, 4 osimertinib-resistant) and 6 surgically resected tumors. Samples were grouped as “osimertinib-sensitive” or “osimertinib-resistant,” with sample type included as a covariate. Data were downloaded using GEOquery in R (v4.3.x). For microarray data, quantile normalization was applied using limma; for RNA-seq data, low-expression genes were removed, followed by TMM normalization in edgeR and voom transformation in limma. Differential expression was analyzed using a linear model (limma) with FDR adjustment by the Benjamini–Hochberg method. Genes with |log₂ fold change | ≥ 1 and FDR < 0.05 were considered significant. A volcano plot was generated with ggplot2, highlighting YAP1 among differentially expressed genes.

### Mass spectrometric identification of MST1 palmitoylation

Endogenous MST1 was immunoprecipitated from cell lysates and subjected to Acyl-RAC to enrich S-palmitoylated proteins. Briefly, proteins were reduced, alkylated, treated with hydroxylamine to cleave thioester bonds, and the newly exposed cysteines were captured on thiol-reactive resin. After elution, MST1 was digested with trypsin and analyzed by nanoLC–MS/MS (EASY-nLC 1200 coupled to an Orbitrap Fusion Lumos). Mass spectra were searched against the UniProt human database using MaxQuant with carbamidomethyl-Cys as a fixed modification and oxidation-Met and palmitoylation-Cys as variable modifications. A palmitoylated peptide THNCWVLEGIIIPNRVCAR was identified with the modification site localized to Cys699.

### Statistical analysis

The investigator was blinded to the group allocation during the experiment. The data were derived from at least three independent experiments and presented as mean ± SD. The difference between the two groups and the multigroup was analyzed using the unpaired t-test and one-way analysis of variance (ANOVA). *P* < 0.05 was statistically significant. *P < 0.05, **P < 0.01 and ***P < 0.001.

## Supplementary information

Supplementary Materials

## Data Availability

All data generated or analysed during this study are included in this published article and its supplementary information files.
