# Supplementary material for: Inhibition of BRD4 sensitizes NSCLC cells to osimertinib by suppressing APT1 and promoting MST1 palmitoylation
Source: Cell Death Discov. 2025 Nov 3;11:497. doi: 10.1038/s41420-025-02794-1 (PMC12583557; doi:10.1038/s41420-025-02794-1)

Supplementary Materials

Supplementary Figure and Figure legends.

Supplementary Figure 1. Generation and characterization of osimertinib-resistant NSCLC cell lines.

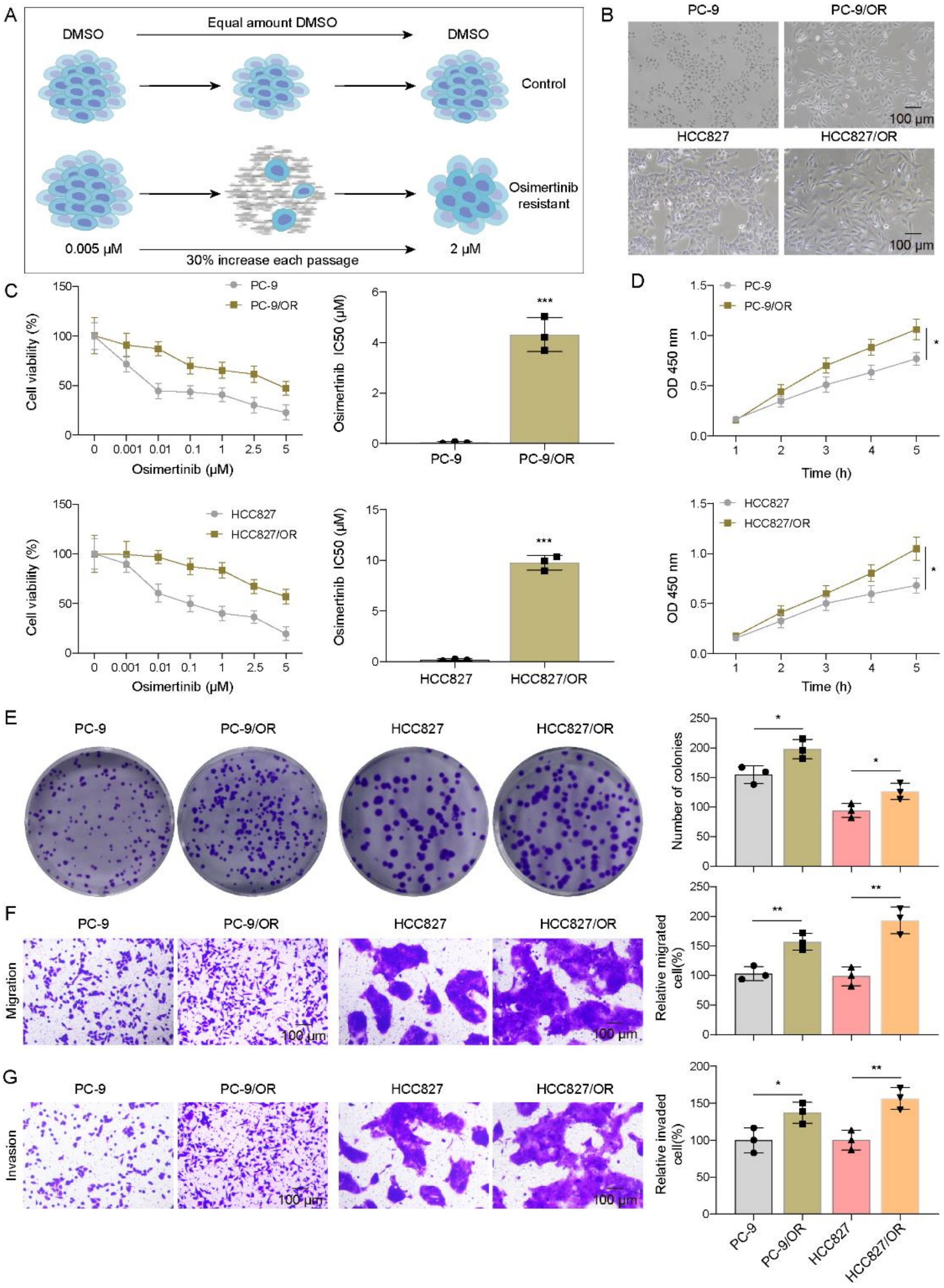

(A) Schematic diagram of the dose-escalation method used to generate osimertinib-resistant PC-9/OR and HCC827/OR cells from parental PC-9 and HCC827 cells. (B)

Morphological comparison of parental and resistant cells observed by phase-contrast microscopy, Scale bar = 100 μm. (C) Cell viability assays and IC_{50} determination following osimertinib treatment in parental and resistant cells. (D) CCK-8 proliferation assays were conducted to detect parental and resistant cell viability. (E) Colony formation assay performed to assess proliferative capacity. (F) Transwell migration assays of parental and resistant cells assessed cell migration, Scale bar = 100 μm. (G) Transwell invasion assays of parental and resistant cells examined cell invasion, Scale bar = 100 μm. Mean ± SD, n = 3, **p* < 0.05, ***p* < 0.01, ****p* < 0.001.

Supplementary Figure 2. YAP1 knockdown reduced YAP1 expression in both cytoplasm and nucleus of osimertinib-resistant cells.

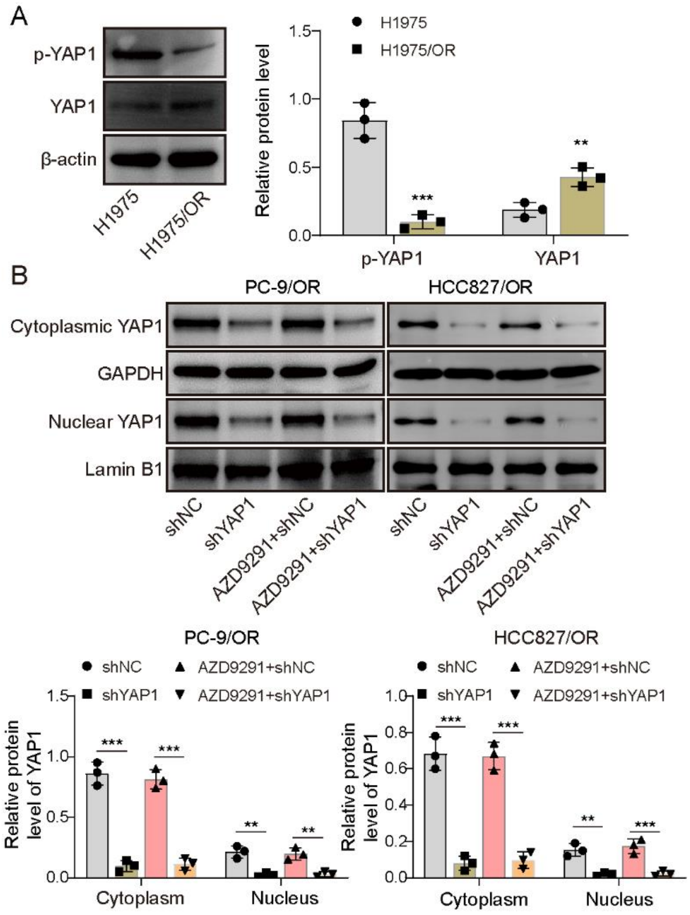

(A) Western blot analysis of p-YAP1 and YAP1 in H1975 and H1975/OR cells. (B) Western blot analysis of cytoplasmic and nuclear YAP1 in PC-9/OR and HCC827/OR

cells with shYAP1 and/or AZD9291 treatment. Mean ± SD, n = 3, $**p < 0.01$, $***p < 0.001$.

Supplementary Figure 3. Overexpression of YAP1 inhibited osimertinib sensitivity.

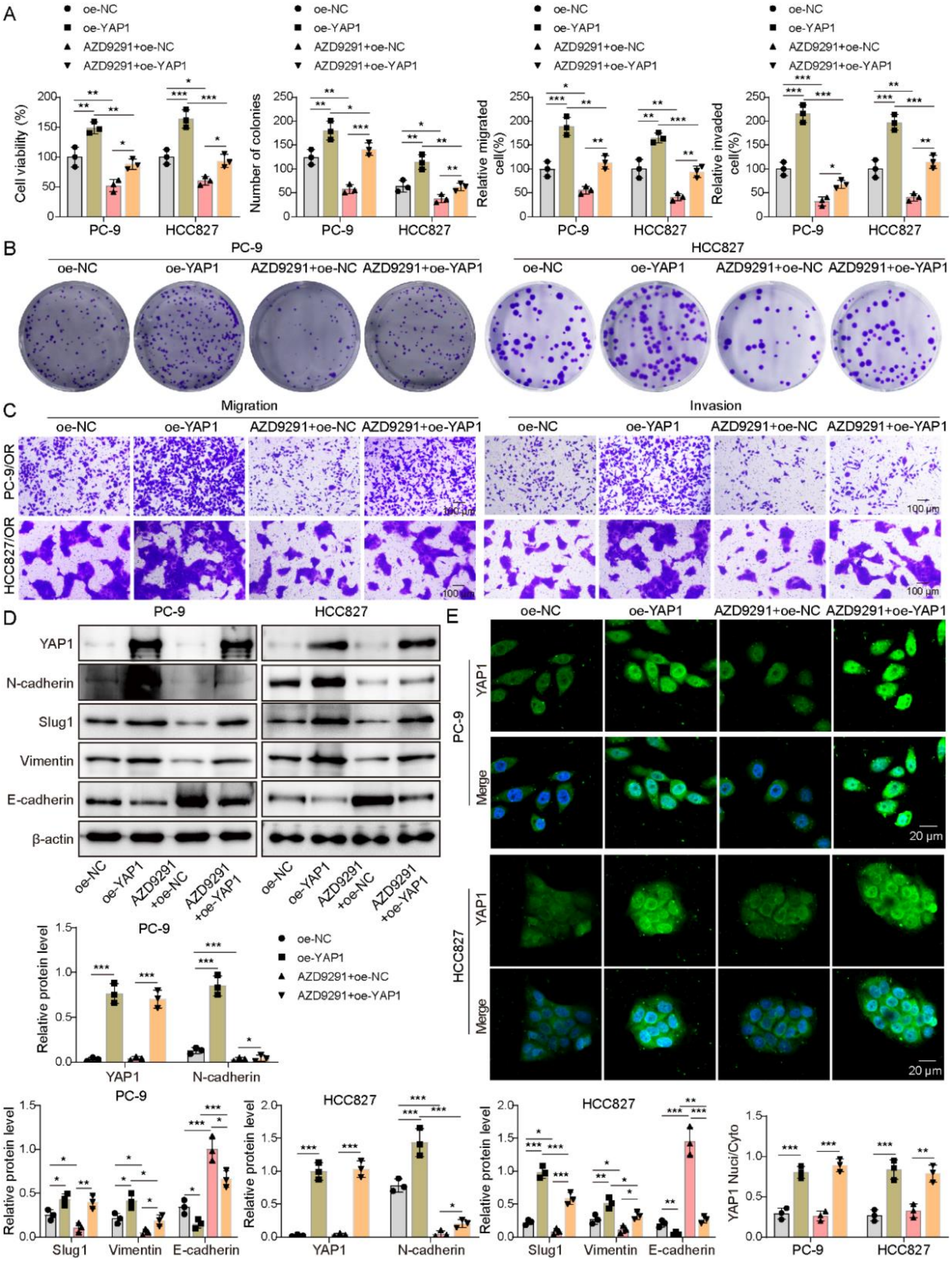

YAP1 was overexpressed in PC-9 and HCC827 cells which were treated with

AZD9291 at 0.1 μM for 24 h and divided into oe-NC, oe-YAP1, AZD9291 + oe-NC and AZD9291 + oe-YAP1 groups. (A) Cell viability. (B) Colony formation assay. (C) Cell migration and invasion, Scale bar = 100 μm. (D) YAP1, N-cadherin, Slug1, Vimentin and E-cadherin were detected by Western blotting. (E) The localization of YAP1 was examined by IF staining. Scale bar, 20 μm. Mean ± SD, n = 3, *$p < 0.05$, **$p < 0.01$, ***$p < 0.001$.

Supplementary Figure 4. BRD4 is upregulated and BRD2/3 don't interact with YAP1 in osimertinib-resistant cells.

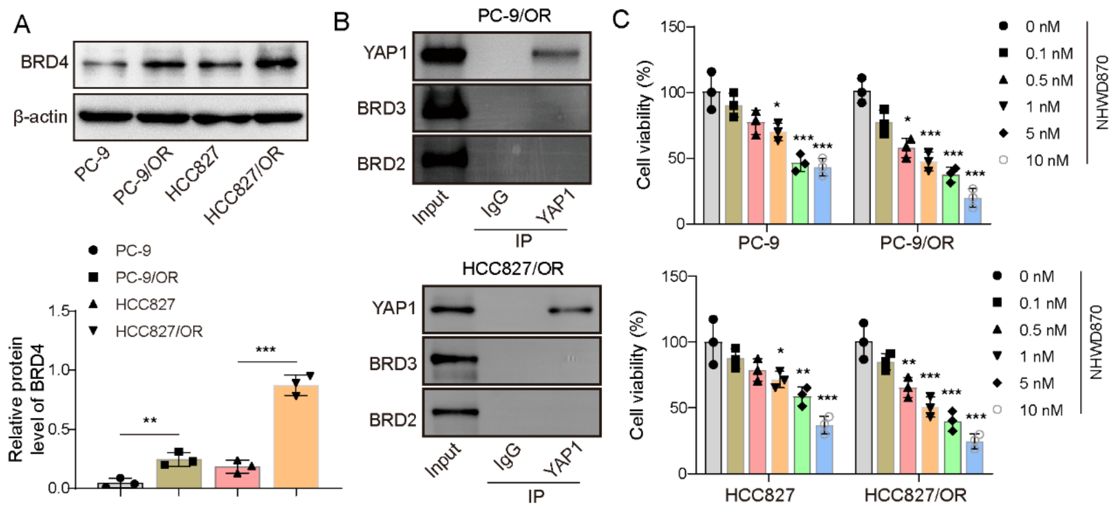

(A) Western blot analysis of BRD4 in parental (PC-9 and HCC827) and resistant cells (PC-9/OR and HCC827/OR). (B) Co-IP detected the interaction between YAP and BRD2 or BRD3. (C) CCK-8 proliferation assays were conducted to detect cell viability in parental and resistant cells treated with NHWD870 at 0, 0.1, 0.5, 1, 5, or 10 nM. Mean ± SD, n = 3, *$p < 0.05$, **$p < 0.01$, ***$p < 0.001$.

Supplementary Figure 5. The BRD4 inhibitor significantly reversed YAP1-mediated osimertinib resistance in NSCLC cells.

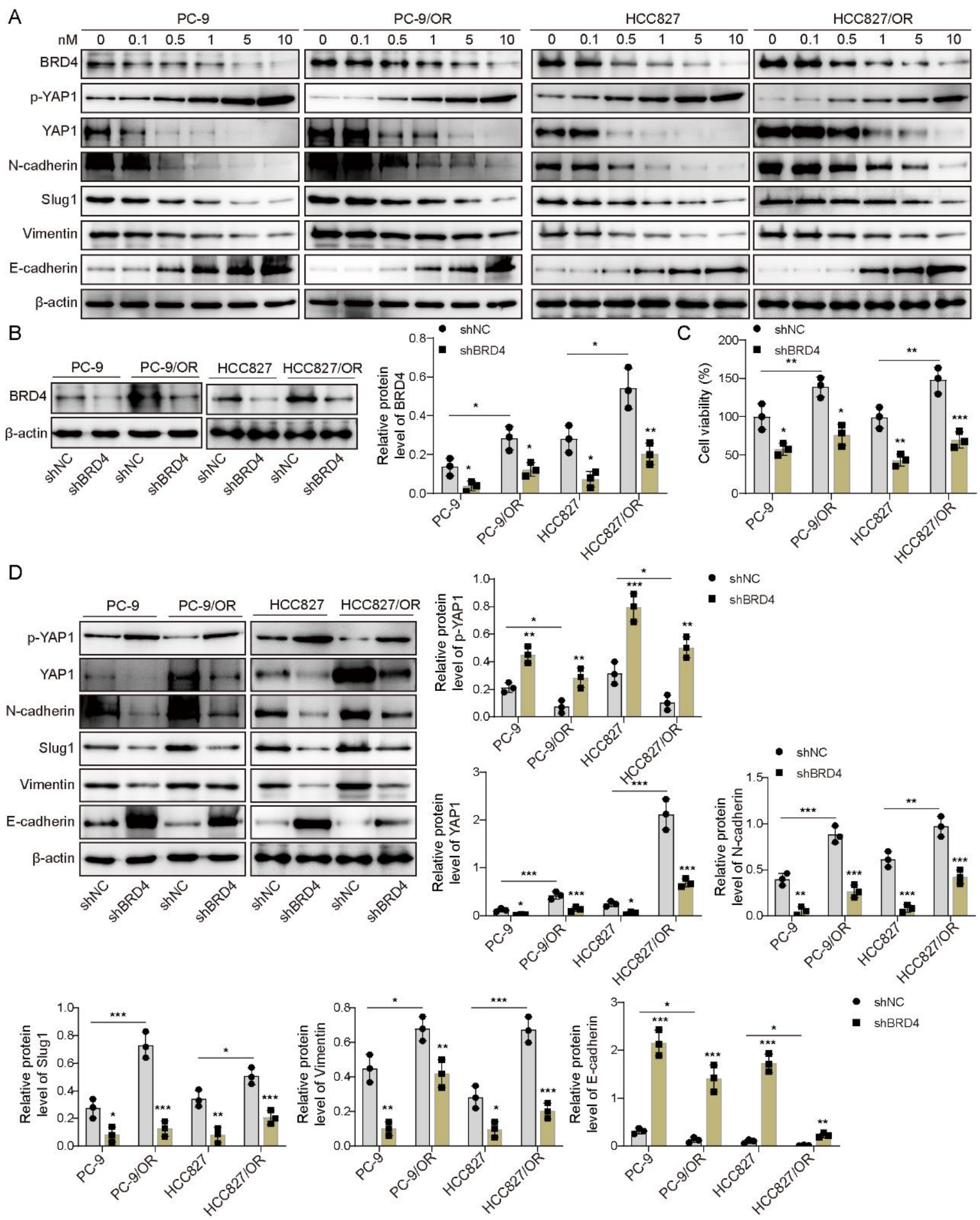

Cells were treated with NHWD870 at 0, 0.1, 0.5, 1, 5, or 10 nM. (A) Western blotting analysis of BRD4, p-YAP1, YAP1, N-cadherin, Slug1, Vimentin and E-cadherin. BRD4 was knocked down, and cells were divided into shNC and shBRD4 groups. (B) BRD4 was detected by Western blotting. (C) Cell viability. (D) Western blotting analysis of p-YAP1, YAP1, N-cadherin, Slug1, Vimentin and E-cadherin. Mean ± SD, n = 3, *$p < 0.05$, **$p < 0.01$, ***$p < 0.001$.

Supplementary Figure 6. AZD9291 and inhibiting BRD4 cooperatively inhibited malignant behaviors and EMT in parental NSCLC cells.

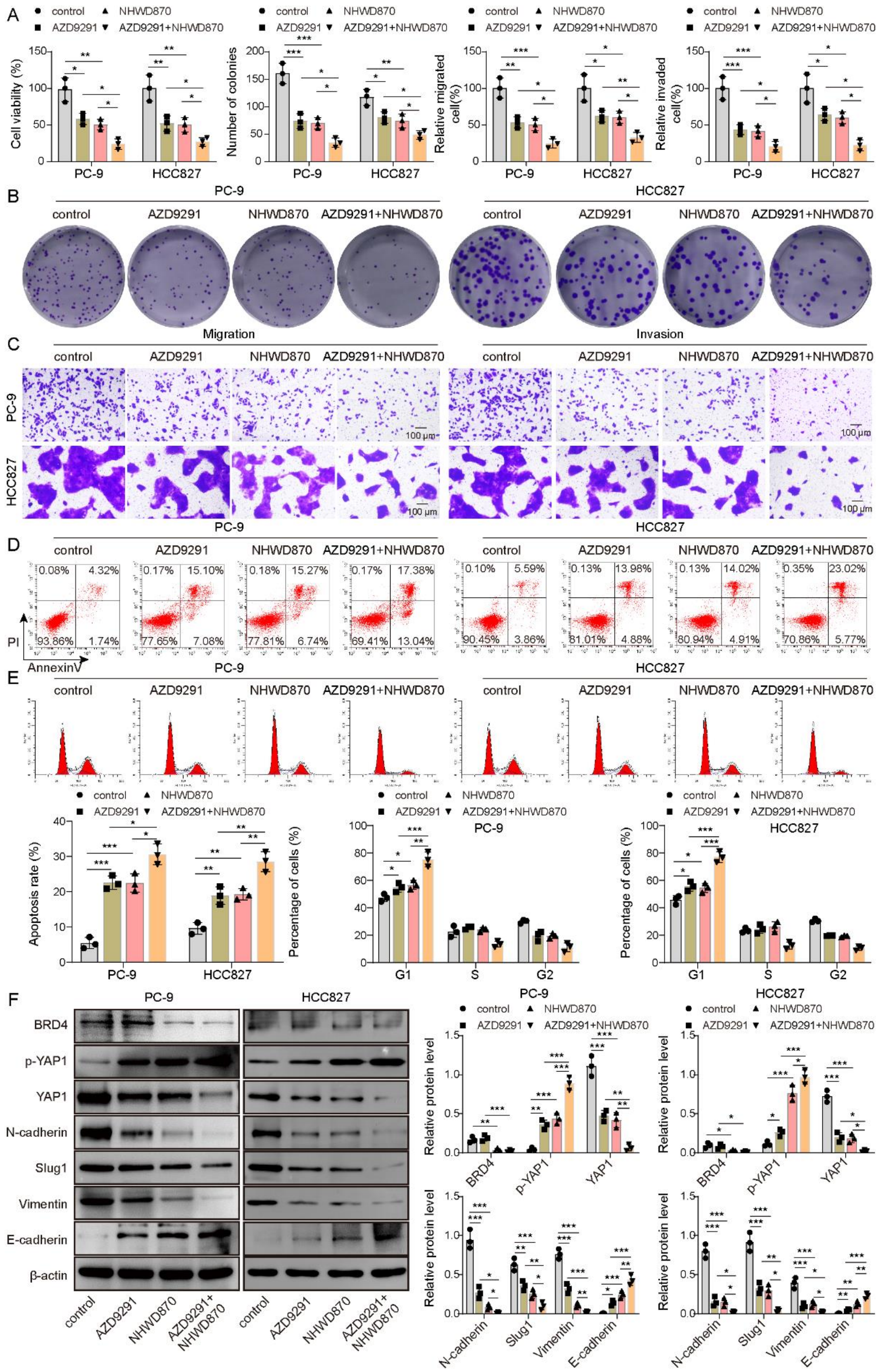

(A) Cell viability was evaluated via CCK-8 assays in PC-9 and HCC827 cells treated with vehicle control, AZD9291, NHWD870, or a combination. (B) Representative images from colony formation assays under each treatment condition. (C) Representative images of transwell migration and invasion assays for treated PC-9 and HCC827 cells, with quantification in bar graphs, Scale bar = 100 μm. (D) Flow cytometric analysis of apoptosis by Annexin V–FITC/PI staining in treated groups; representative plots and percentage of apoptotic cells shown. (E) Cell cycle distribution profiles determined via flow cytometry with quantification of G1, S, and G2 phase populations in treated cells. (F) Western blot analysis of BRD4, p-YAP1, active YAP1, and EMT-related markers (N-cadherin, Slug1, Vimentin, E-cadherin) in cells treated with AZD9291, NHWD870, or their combination. Bar graphs display quantified relative protein expression levels. Mean ± SD, n = 3, *$p < 0.05$, **$p < 0.01$, ***$p < 0.001$.

Supplementary Figure 7. BRD4 inhibition enhanced AZD9291 sensitivity in NSCLC cells.

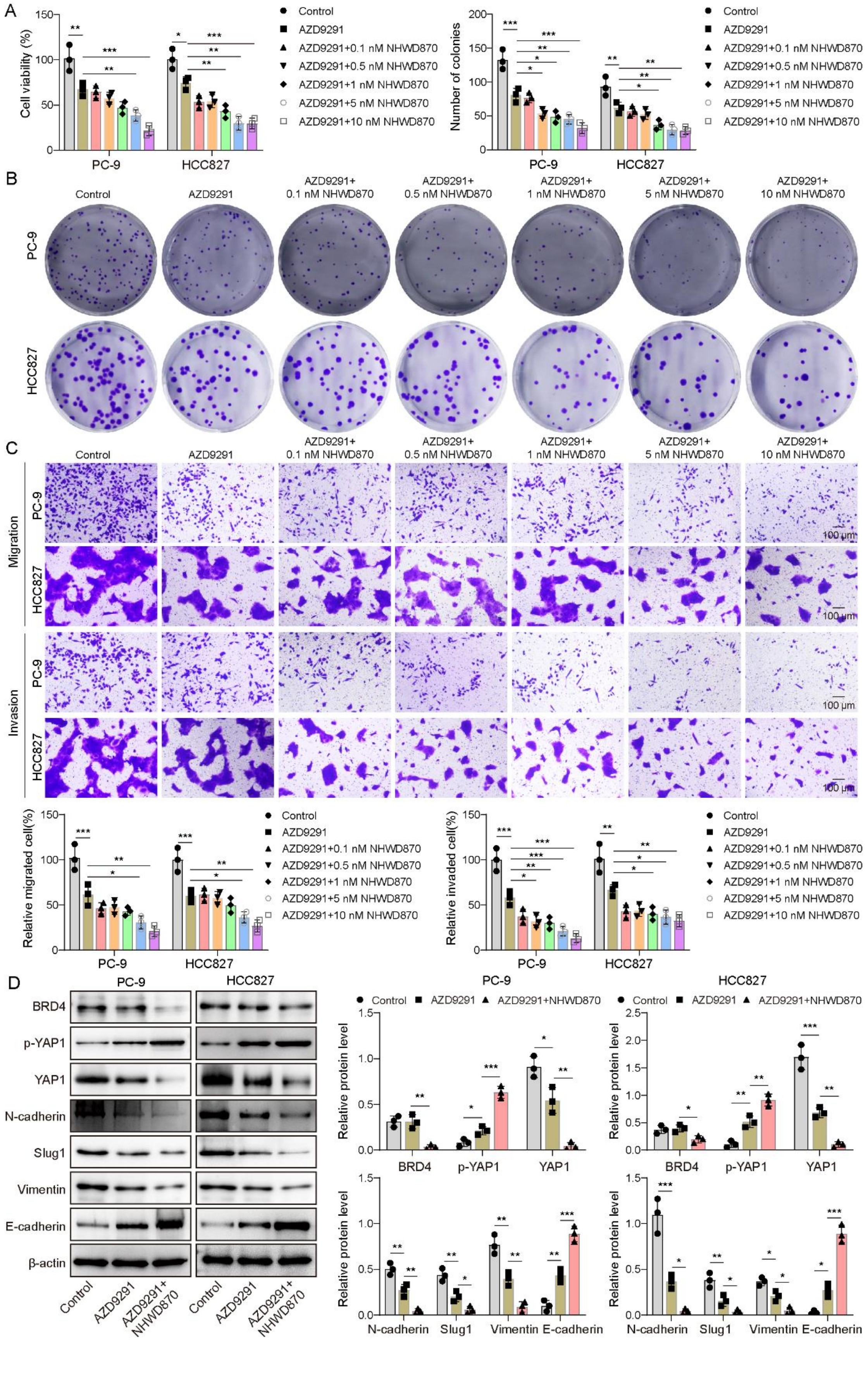
A
Cell viability (%)
PC-9
HCC827
Number of colonies
Control
AZD9291
AZD9291+0.1 nM NHWD870
AZD9291+0.5 nM NHWD870
AZD9291+1 nM NHWD870
AZD9291+5 nM NHWD870
AZD9291+10 nM NHWD870
B
Control
AZD9291
AZD9291+ 0.1 nM NHWD870
AZD9291+ 0.5 nM NHWD870
AZD9291+ 1 nM NHWD870
AZD9291+ 5 nM NHWD870
AZD9291+ 10 nM NHWD870
PC-9
HCC827
C
Migration
Invasion
100 μm
Relative migrated cell(%)
Relative invaded cell(%)
D
PC-9
HCC827
BRD4
p-YAP1
YAP1
N-cadherin
Slug1
Vimentin
E-cadherin
β-actin
Control
AZD9291
AZD9291+ NHWD870
AZD9291+NHWD870
Relative protein level

(A) Cell viability of PC-9 and HCC827 cells treated with AZD9291 alone or in combination with NHWD870 at various doses (0, 0.1, 0.5, 1, 5, or 10 nM). (B) Colony formation assays of PC-9 and HCC827 cells following treatment with AZD9291 and/or NHWD870. (C) Transwell migration (top) and invasion (bottom) assays of PC-9 and HCC827 cells and quantification of migrated and invaded cells shown in bar graphs, Scale bar = 100 μm. (D) Western blot analysis of BRD4, active YAP1, phosphorylated YAP1 (p-YAP1), and EMT-related proteins (N-cadherin, Slug1, Vimentin, E-cadherin) in PC-9 and HCC827 cells under indicated treatments; bar graphs show quantification of relative protein expression. Mean ± SD, n = 3, *$p < 0.05$, **$p < 0.01$, ***$p < 0.001$.

Supplementary Figure 8. BRD4 inhibition suppressed YAP1 overexpression-driven malignant phenotypes in NSCLC cells.

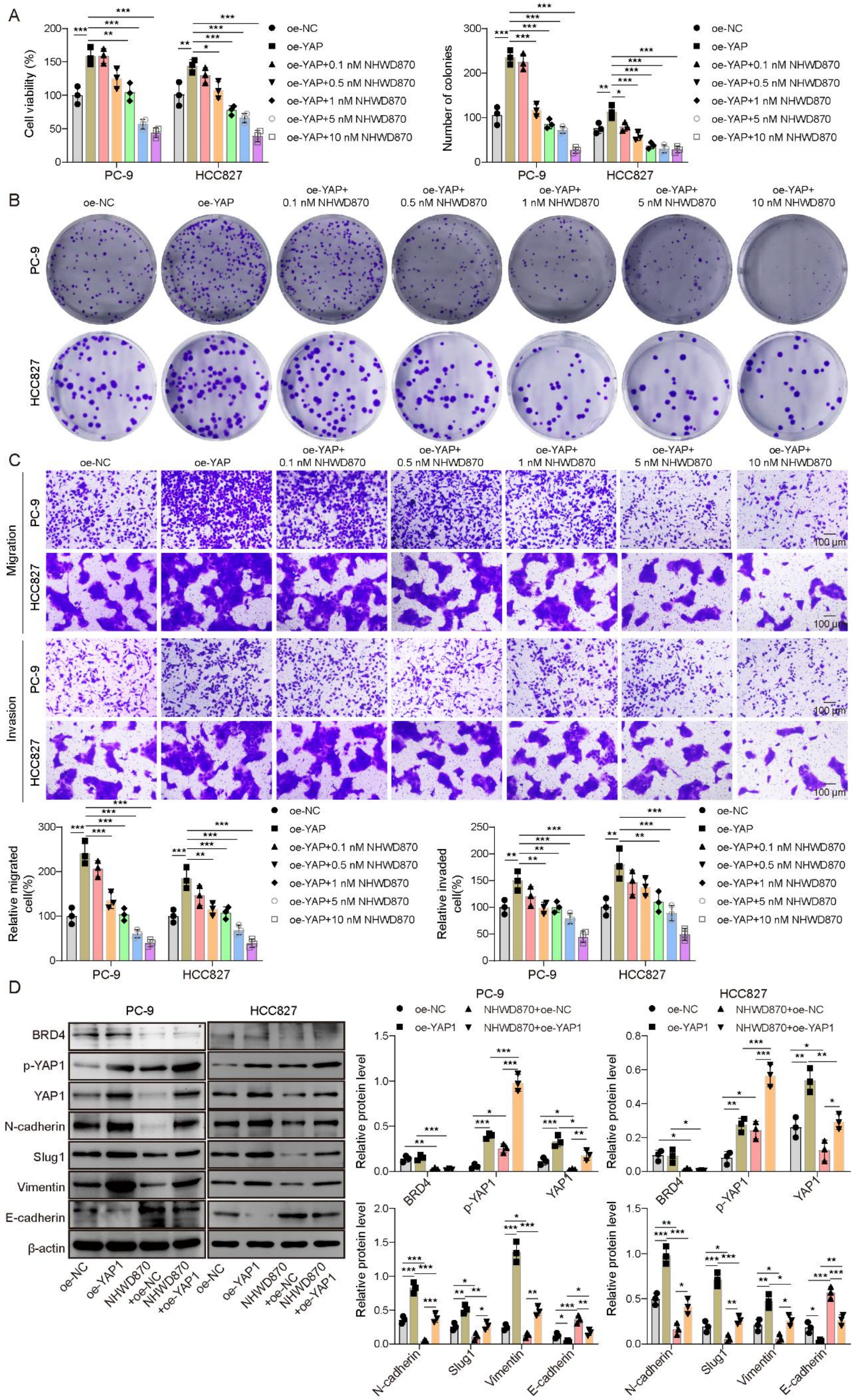

A
Cell viability (%)
PC-9
HCC827
Number of colonies
oe-NC
oe-YAP
oe-YAP+0.1 nM NHWD870
oe-YAP+0.5 nM NHWD870
oe-YAP+1 nM NHWD870
oe-YAP+5 nM NHWD870
oe-YAP+10 nM NHWD870
B
oe-NC
oe-YAP
oe-YAP+ 0.1 nM NHWD870
oe-YAP+ 0.5 nM NHWD870
oe-YAP+ 1 nM NHWD870
oe-YAP+ 5 nM NHWD870
oe-YAP+ 10 nM NHWD870
PC-9
HCC827
C
Migration
Invasion
100 μm
Relative migrated cell(%)
Relative invaded cell(%)
D
PC-9
HCC827
BRD4
p-YAP1
YAP1
N-cadherin
Slug1
Vimentin
E-cadherin
β-actin
oe-NC
oe-YAP1
NHWD870 +oe-NC
NHWD870 +oe-YAP1
NHWD870+oe-NC
NHWD870+oe-YAP1
Relative protein level

(A) Cell viability of PC-9 and HCC827 cells overexpressing YAP1 (oe-YAP1) or control vector (oe-NC) following treatment with increasing concentrations (0, 0.1, 0.5, 1, 5, or 10 nM) of the BRD4 inhibitor NHWD870. (B) Colony formation assays of PC-9 and HCC827 cells following treatment with NHWD870 (0, 0.1, 0.5, 1, 5, or 10 nM) on YAP1-overexpressing PC-9 and HCC827 cells. (C) Representative images and quantification of transwell migration and invasion assays under the same conditions as (B), scale bar = 100 μm. (D) Western blot analysis of BRD4, active YAP1, phosphorylated YAP1 (p-YAP1), and EMT markers (N-cadherin, Slug1, Vimentin, and E-cadherin) in oe-YAP1 and oe-NC PC-9 and HCC827 cells treated with NHWD870 (5 nM). Bar graphs show quantified protein levels normalized to β-actin. Mean ± SD, n = 3, *$p < 0.05$, **$p < 0.01$, ***$p < 0.001$.

Supplementary Figure 9. The BRD4 inhibitor blocked YAP1-mediated malignant behaviors and EMT.

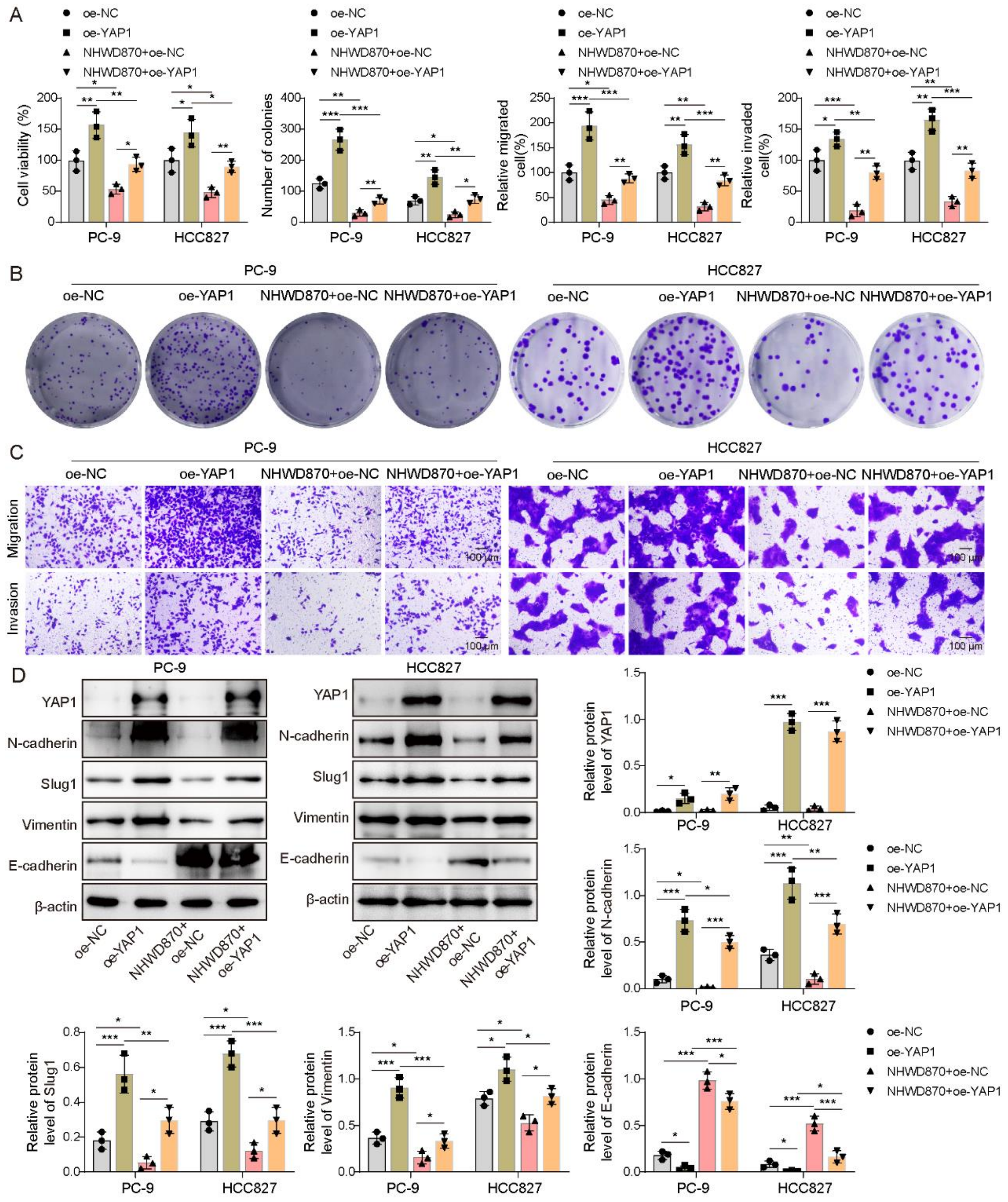

YAP1-overexpressing PC-9 and HCC827 cells were treated with NHWD870 and divided into oe-NC, oe-YAP1, NHWD870+oe-NC, and NHWD870 + oe-YAP1 groups. (A) Cell viability. (B) Colony formation assay. (C) Cell migration and invasion, scale bar = 100 µm. (D) Western blotting analysis of YAP1, N-cadherin, Slug1, Vimentin, and E-cadherin. Mean ± SD, n = 3, *$p < 0.05$, **$p < 0.01$, ***$p < 0.001$.

Supplementary Figure 10. APT1 regulated MST1 palmitoylation and modulated YAP1 signaling in osimertinib-resistant NSCLC cells.

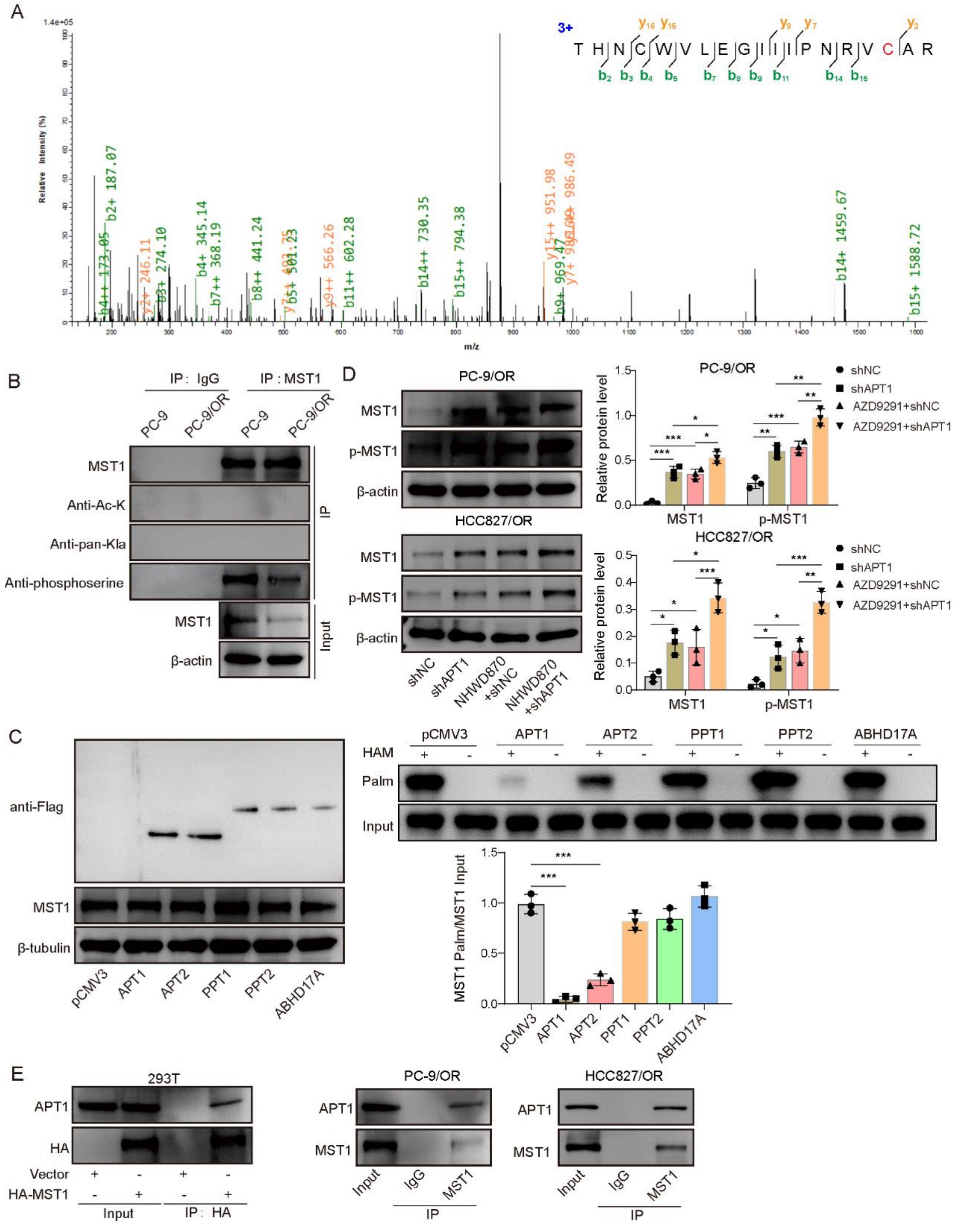

(A) The palmitoylation modification sites of MST1 were identified through mass spectrometry. (B) Co-IP and immunoblotting for acetylation (Ac-K), pan lactylation (pan-Kla), and phosphorylation (p-Ser) of MST1 in PC-9 and PC-9/OR cells. (C) Western blot of MST1 palmitoylation following overexpression of Flag-tagged

depalmitoylases (APT1, APT2, PPT1, PPT2, ABHD17A) and Acyl-RAC assay assessing palmitoylation of MST1. (D) Western blot analysis of total and phosphorylated MST1 (p-MST1) in PC-9/OR and HCC827/OR cells following shAPT1 or NHWD870 treatment. (E) Co-IP analysis of the interaction between MST1 and APT1 in HEK293T, PC-9/OR, and HCC827/OR cells. Mean ± SD, n = 3, *$p < 0.05$, **$p < 0.01$, ***$p < 0.001$.

Supplementary Figure 11. Inhibiting APT1-mediated MST1 depalmitoylation recovered osimertinib sensitivity.

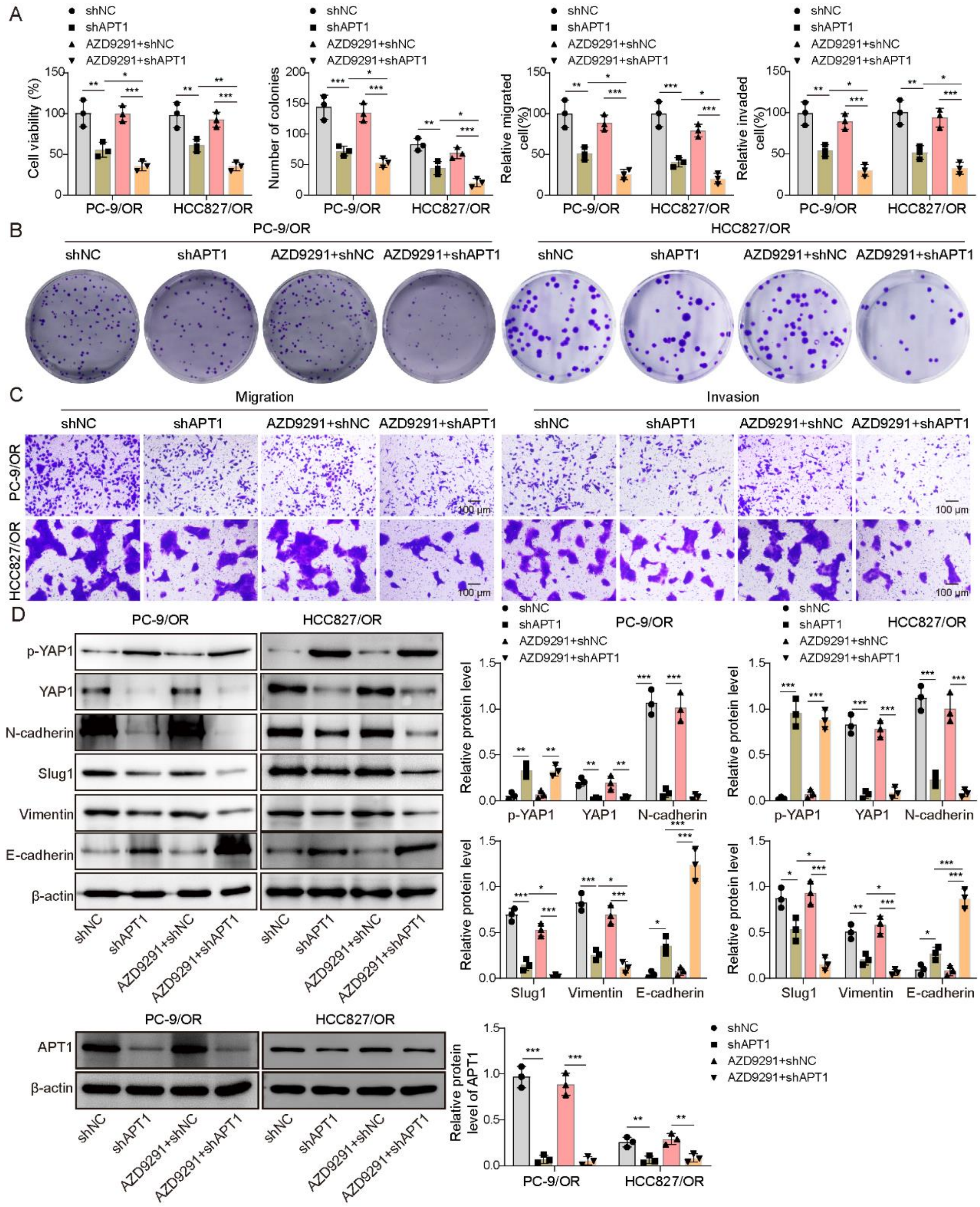

APT1-knockdown cells were treated with AZD9291 and divided into shNC, shAPT1, AZD9291, and shAPT1 + AZD9291 groups. (A) Cell viability. (B) Colony formation assay was applied to assess cell proliferation. (C) Cell migration and invasion, scale bar = 100 μm. (D) Western blotting analysis of APT1, p-YAP1, active YAP1, N-cadherin, Slug1, Vimentin and E-cadherin. Mean ± SD, n = 3, $*p < 0.05$, $**p < 0.01$, $***p < 0.001$.

Supplementary Figure 12. Effects of osimertinib, YAP1, BRD4, and APT1 modulation on AKT signaling in NSCLC cells.

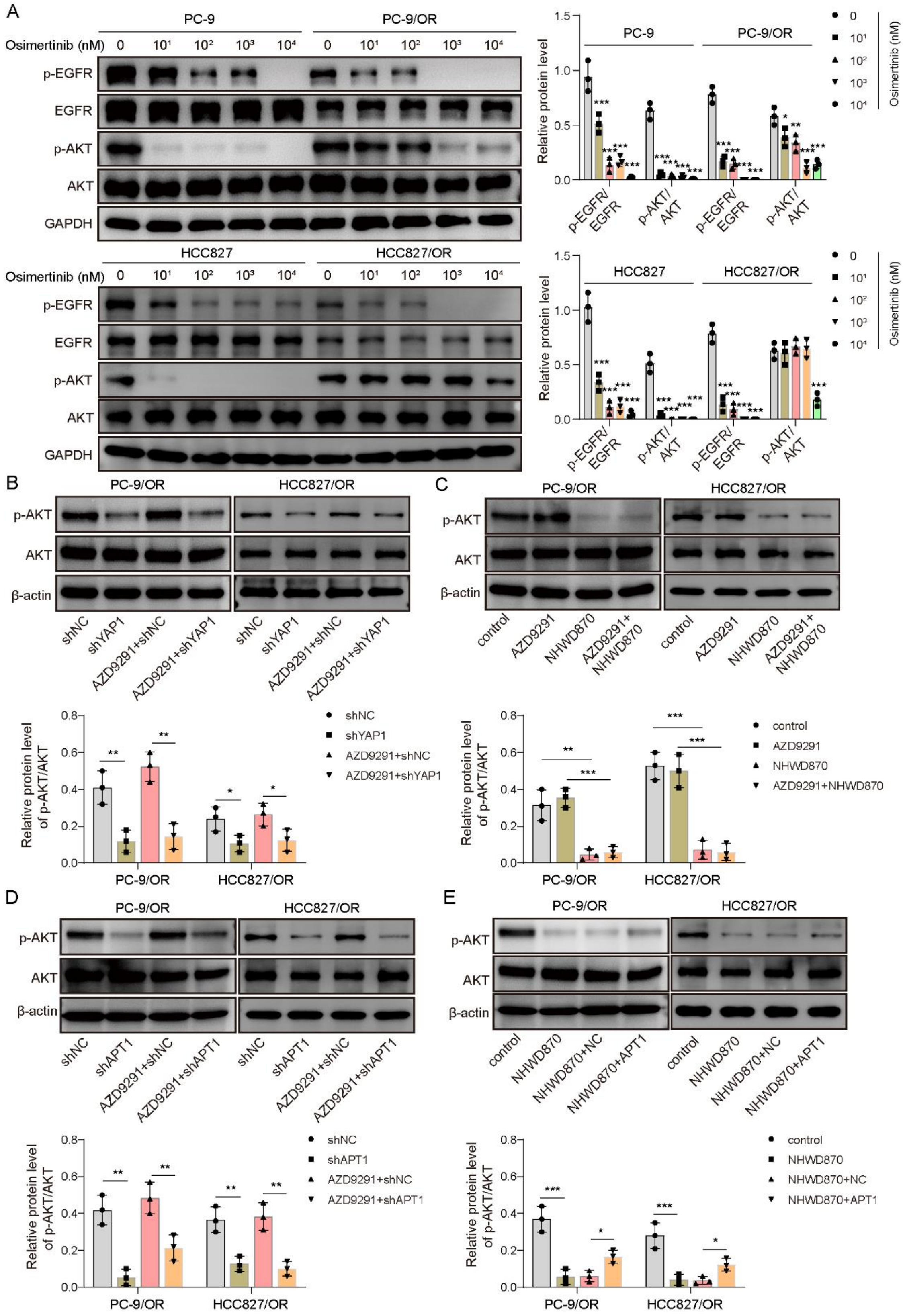

(A) Western blot analysis of p-EGFR, EGFR, p-AKT, and AKT expression in PC-9, PC-9/OR, HCC827, and HCC827/OR cells treated with increasing concentrations of osimertinib (AZD9291: 0, 10^1, 10^2, 10^3, and 10^4 nM). (B) Protein expression of AKT, and p-AKT in PC-9/OR and HCC827/OR cells following shYAP1 knockdown with or without AZD9291 (10^2 nM) treatment. (C) AKT signaling changes in PC-9/OR and HCC827/OR cells treated with AZD9291, NHWD870, or the combination. (D) Western blot analysis of AKT pathway markers in cells transfected with shAPT1 and treated with AZD9291. (E) AKT signaling in PC-9/OR and HCC827/OR cells transfected with overexpressing APT plasmid (oe-APT1) or treated with NHWD870 alone or in combination. Quantification of relative protein levels is shown on the right. Mean ± SD, n = 3, *$p < 0.05$, **$p < 0.01$, ***$p < 0.001$.

Supplementary Figure 13. The BRD4 inhibitor disrupted APT1-mediated depalmitoylation of MST1 to restore osimertinib sensitivity in osimertinib-resistant NSCLC cells.

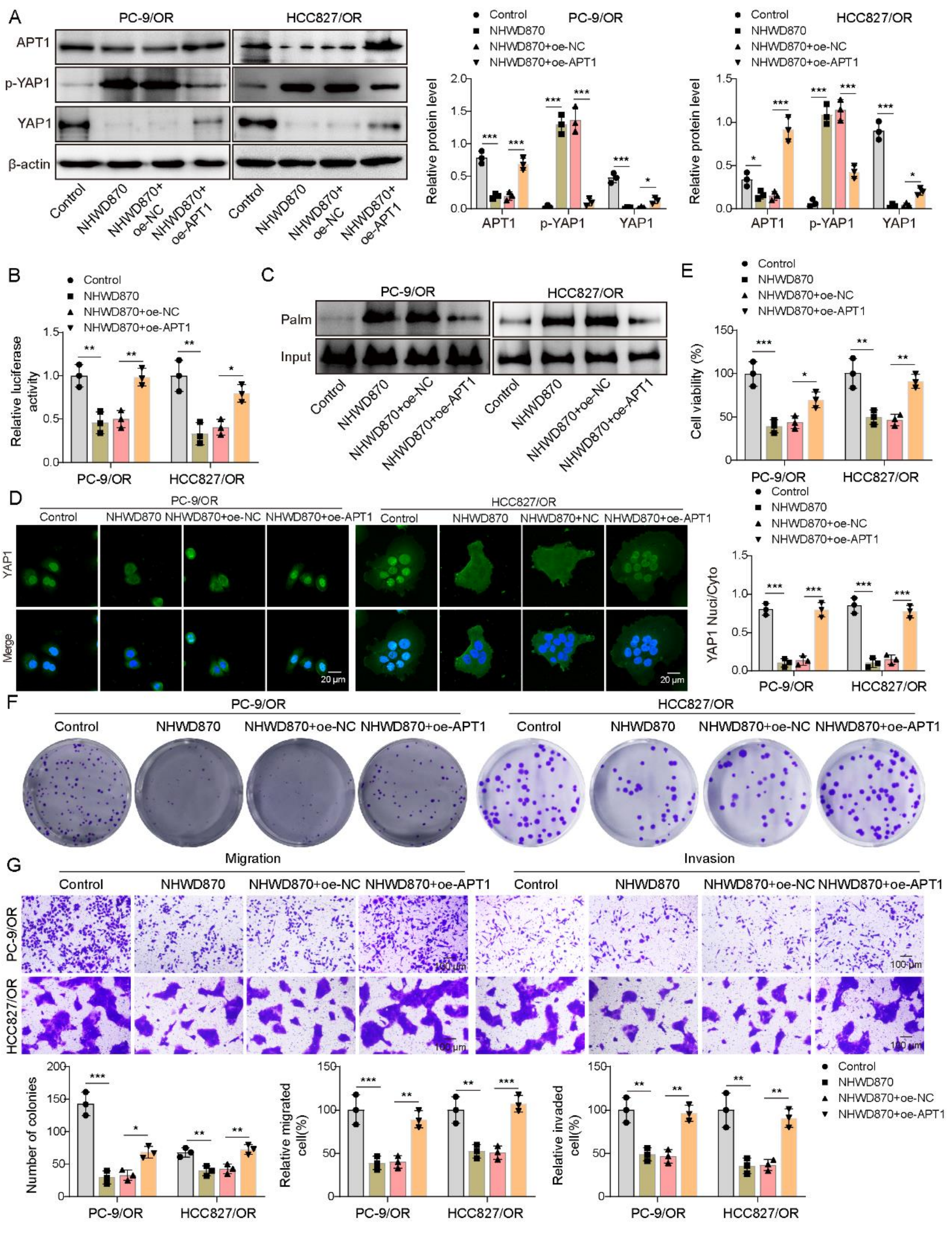

APT1-overexpressing PC-9/OR and HCC827/OR cells were treated with NHWD870 and divided into Control, NHWD870, NHWD870 + oe-NC, and NHWD870 + oe-APT1 groups. (A) The levels of APT1, p-YAP1, and active YAP1 were examined with Western blotting. (B) The activity of the APT1 promoter was evaluated by luciferase activity. (C) MST1 palmitoylation was determined through the Acyl-RAC assay. (D) The localization of YAP1 was examined by IF staining. Scale bar, 20 μm. (E) Cell

viability. (F) Colony formation assay. (G) Cell migration and invasion, scale bar = 100 μm. Mean ± SD, n = 3, *p < 0.05, **p < 0.01, ***p < 0.001.

Supplementary Figure 14. Palmitoylation-deficient MST1 mutant impairs AZD9291-mediated tumor suppression in NSCLC cells.

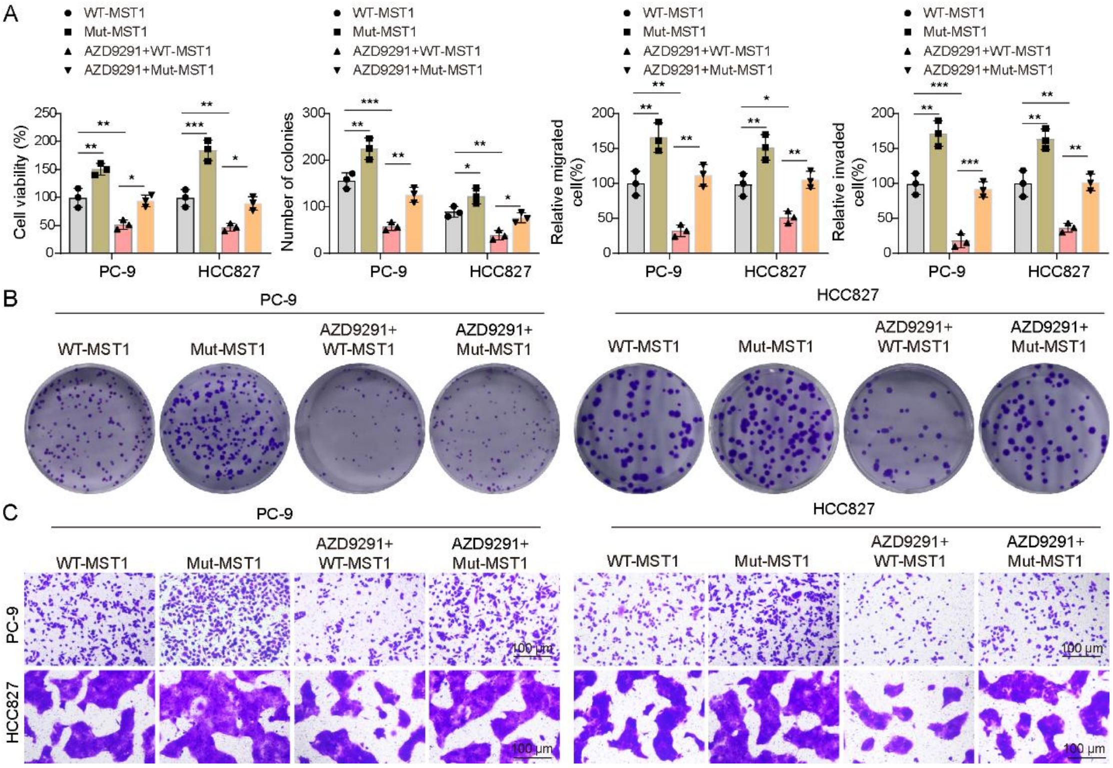

(A) Cell viability, colony formation, migration, and invasion assays were performed in PC-9 and HCC827 cells transfected with wild-type MST1 (WT-MST1) or palmitoylation-deficient mutant MST1 (Mut-MST1), with or without AZD9291 treatment. (B) Representative images of colony formation in the indicated groups. (C) Transwell migration and invasion assays showing reduced suppression by AZD9291 in Mut-MST1-expressing cells compared to WT-MST1, scale bar = 100 μm. Mean ± SD, n = 3, *p < 0.05, **p < 0.01, ***p < 0.001.

Supplementary Figure 15. *In vivo* assessment of AZD9291 and NHWD870 effects in NSCLC xenograft models.

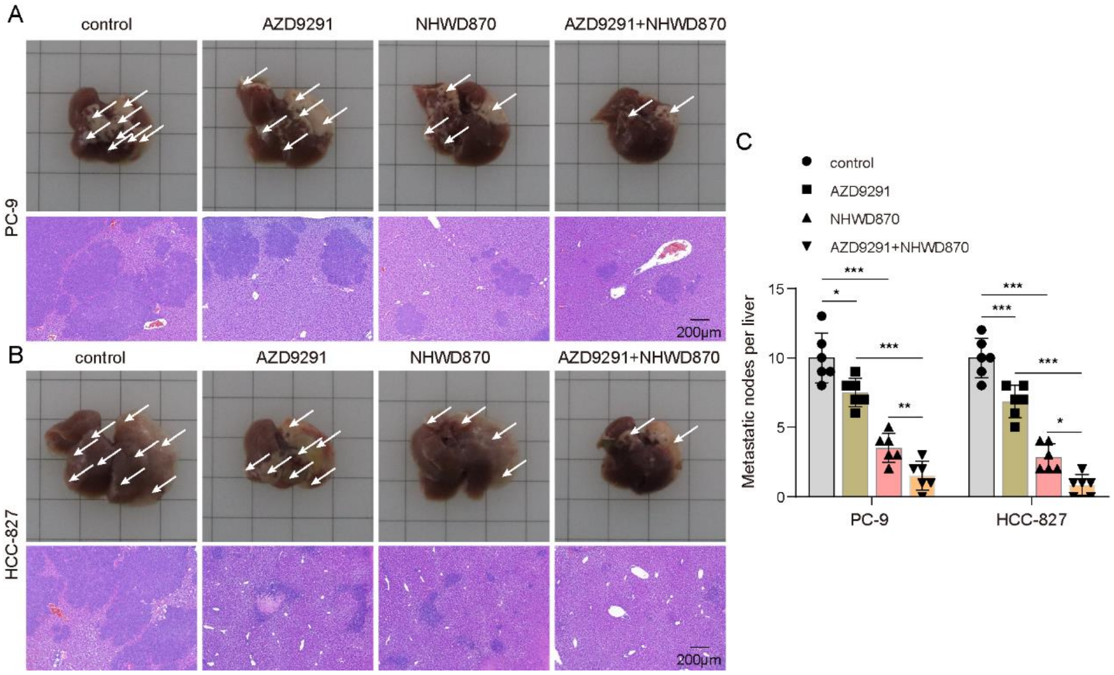

(A-B) Representative images of livers harvested from mice bearing subcutaneous PC-9 or HCC827 tumors after treatment with vehicle control, AZD9291 (1.25 mg/kg), NHWD870 (1.5 mg/kg), or their combination (0.625 mg/kg AZD9291 plus 0.75 mg/kg NHWD870). (C) Quantification of liver tumor nodules following indicated treatments. Mean ± SD, n = 6, *$p < 0.05$, **$p < 0.01$, ***$p < 0.001$.

2. The full and uncropped western blots

1B

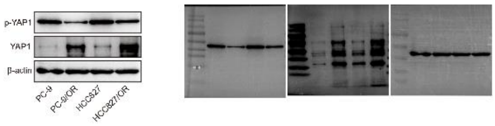

1D

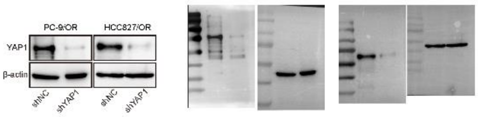

1H

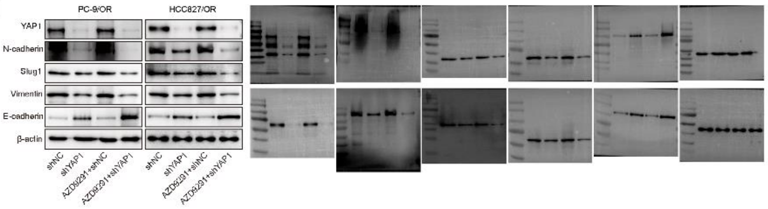

2D

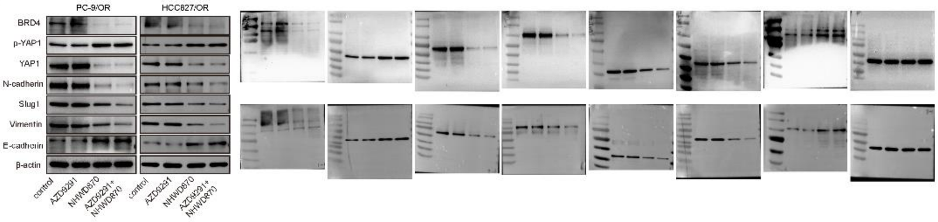

3A

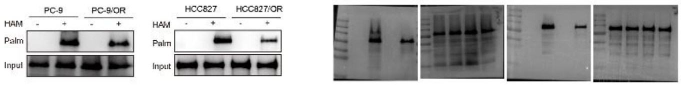

3B

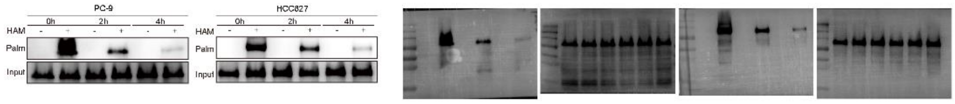

3C

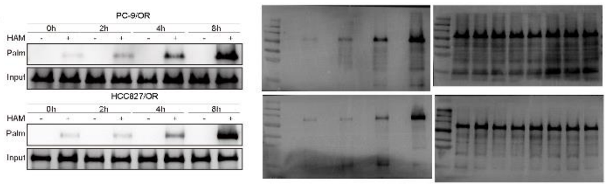

3E

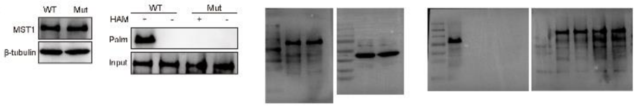

3F

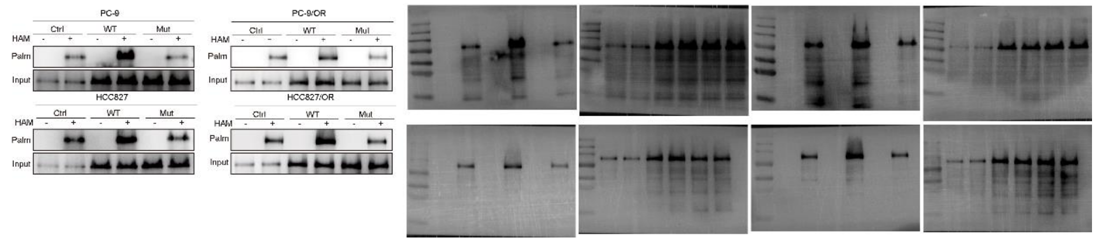

4A

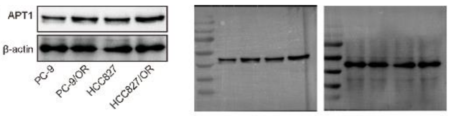
APT1
β-actin
PC-9
PC-9/OR
HCC827
HCC827/OR

4B

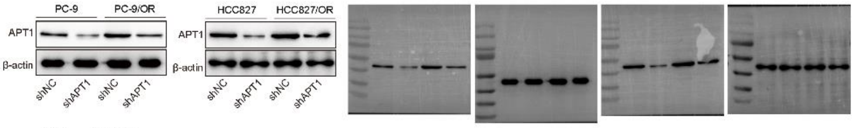
PC-9
PC-9/OR
HCC827
HCC827/OR
APT1
β-actin
shNC
shAPT1

4D

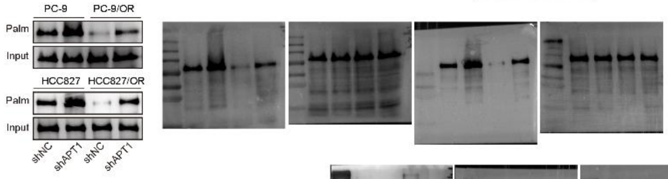
PC-9
PC-9/OR
HCC827
HCC827/OR
Palm
Input
shNC
shAPT1

4F

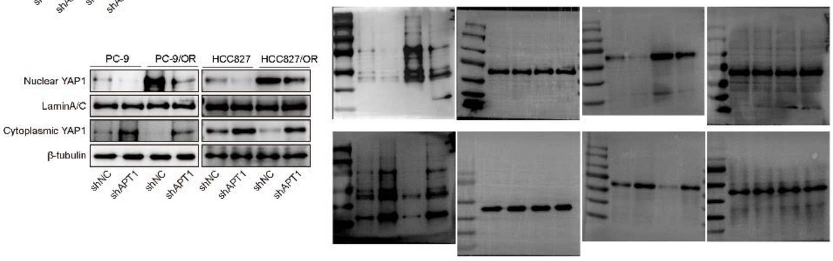
PC-9
PC-9/OR
HCC827
HCC827/OR
Nuclear YAP1
LaminA/C
Cytoplasmic YAP1
β-tubulin
shNC
shAPT1

4G

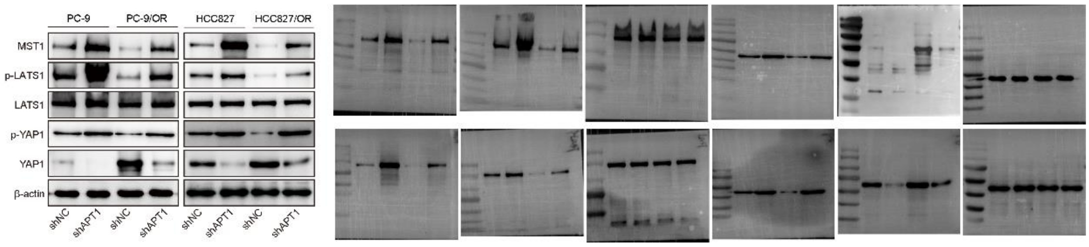
PC-9
PC-9/OR
HCC827
HCC827/OR
MST1
p-LATS1
LATS1
p-YAP1
YAP1
β-actin
shNC
shAPT1

5A

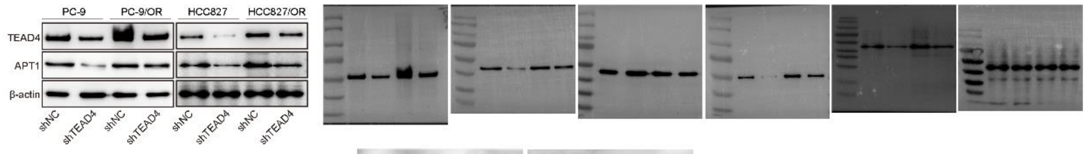

5D

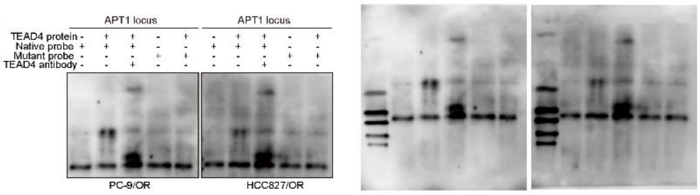

5I

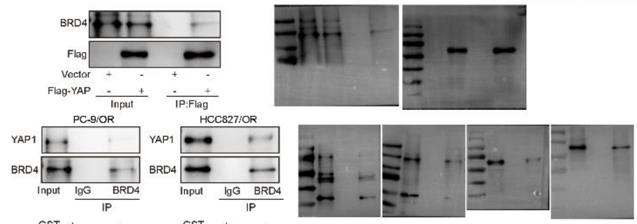

5J

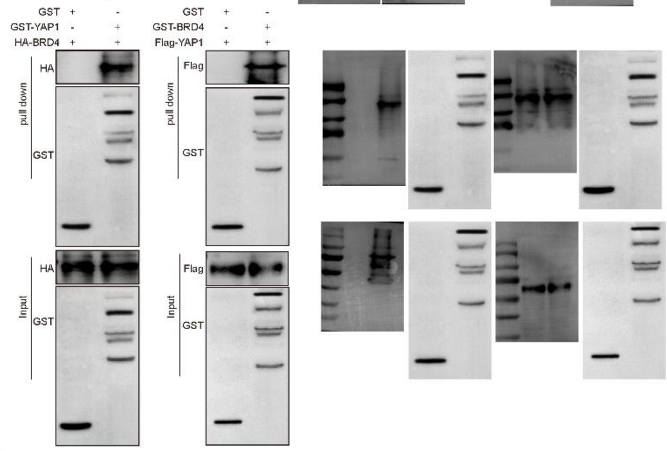

5M

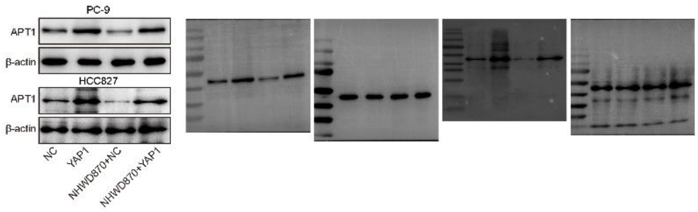

6E

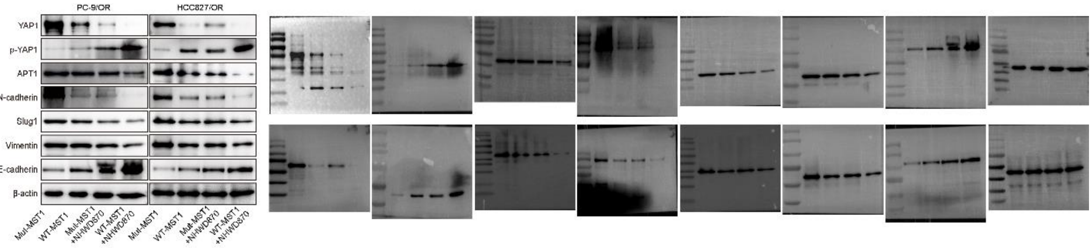

7D

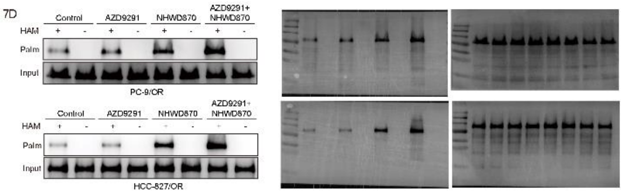

S2A

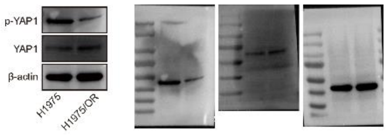

S2B

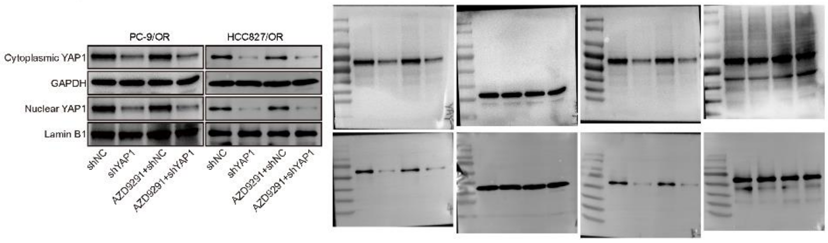

S3D

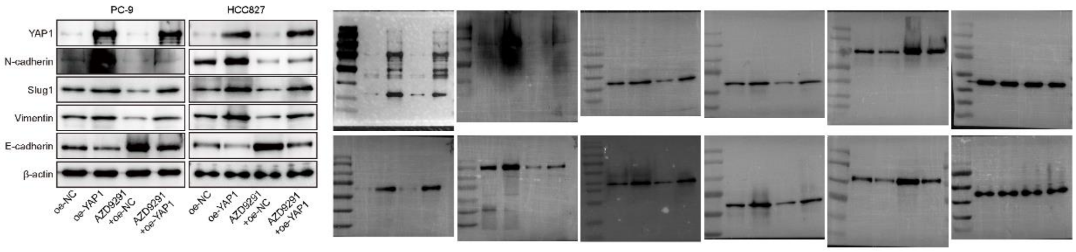

S4A

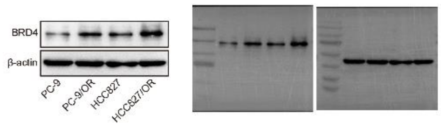

S4B

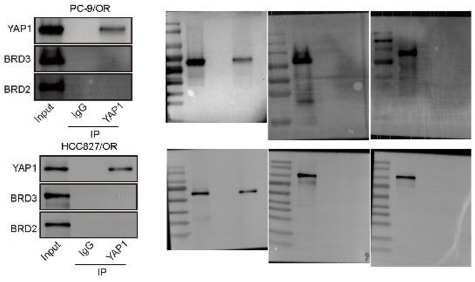

S5A

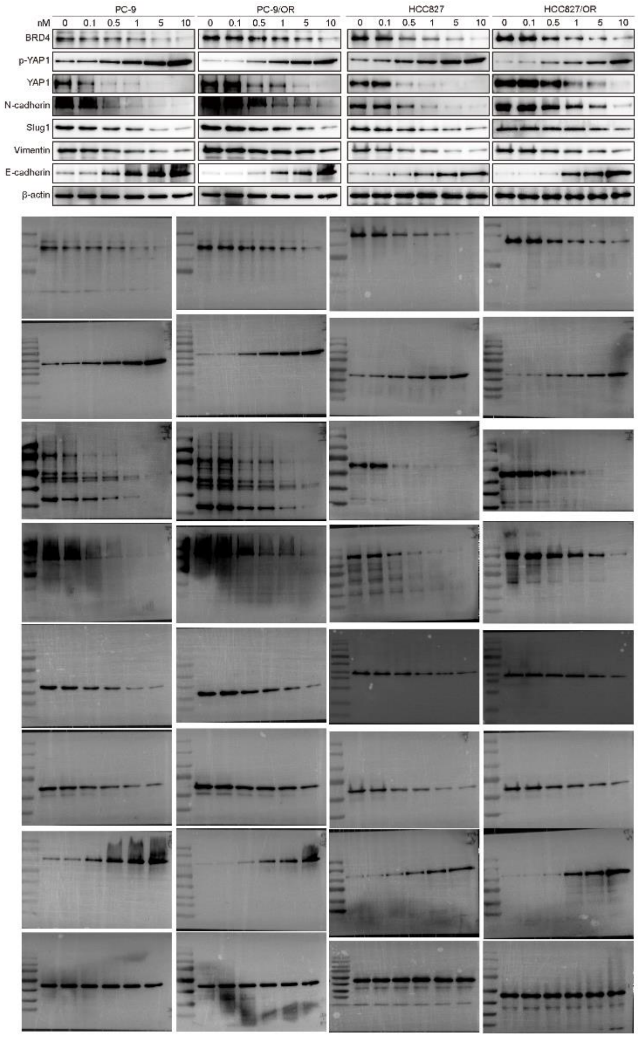
PC-9
PC-9/OR
HCC827
HCC827/OR
nM
0 0.1 0.5 1 5 10
BRD4
p-YAP1
YAP1
N-cadherin
Slug1
Vimentin
E-cadherin
β-actin

S5B

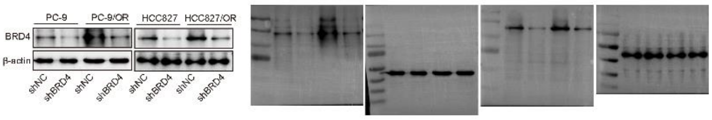
PC-9
PC-9/OR
HCC827
HCC827/OR
BRD4
β-actin
shNC
shBRD4

S5D

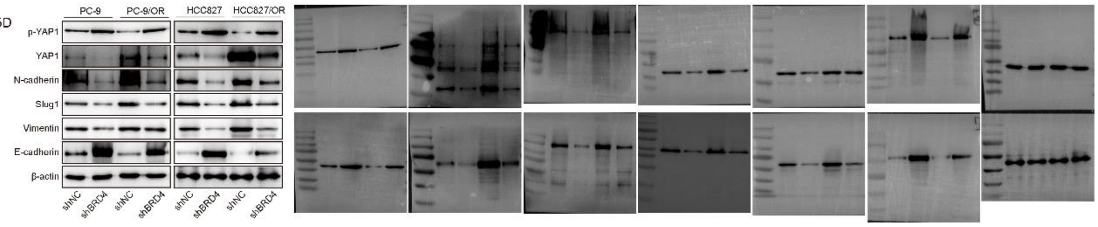
PC-9
PC-9/OR
HCC827
HCC827/OR
p-YAP1
YAP1
N-cadherin
Slug1
Vimentin
E-cadherin
β-actin
shNC
shBRD4

S6F

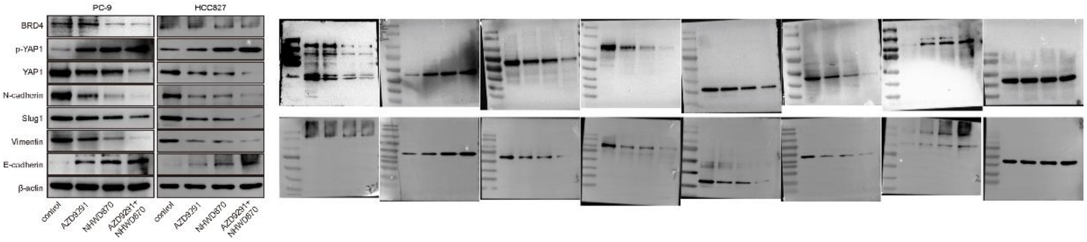

S7D

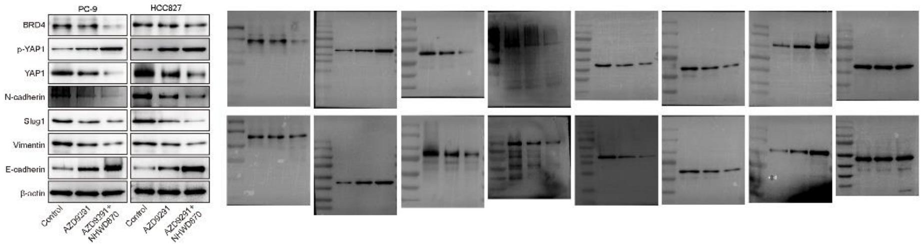

S8D

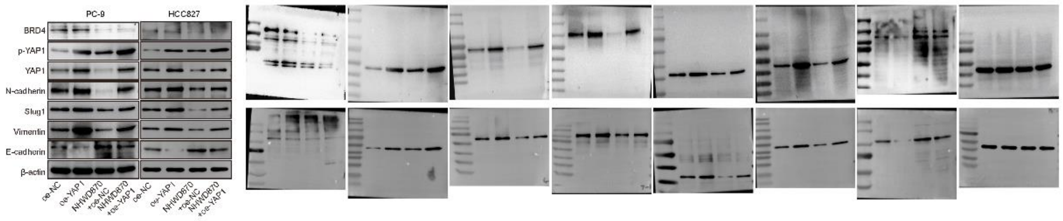

S9B

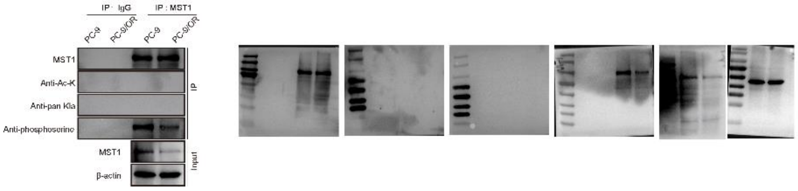

S9C

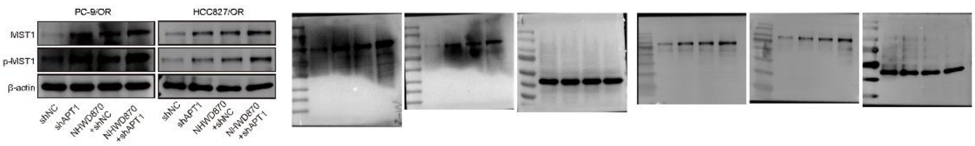

S9D

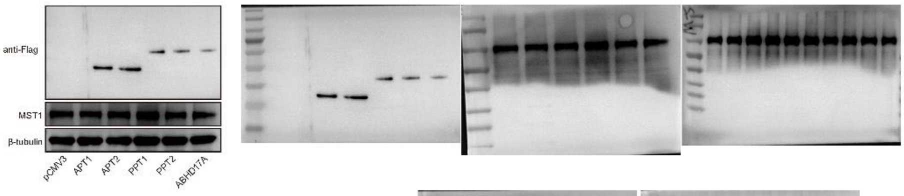

S9E

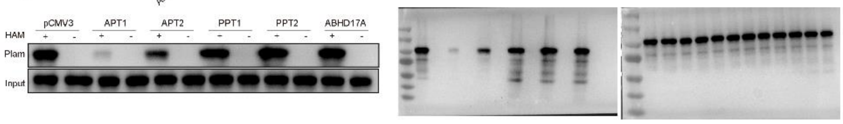

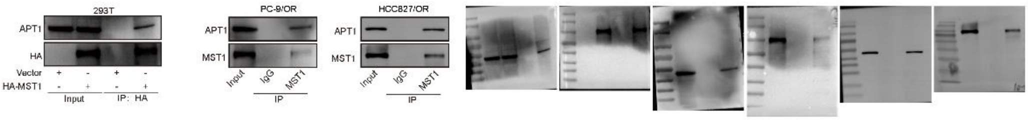

S10D

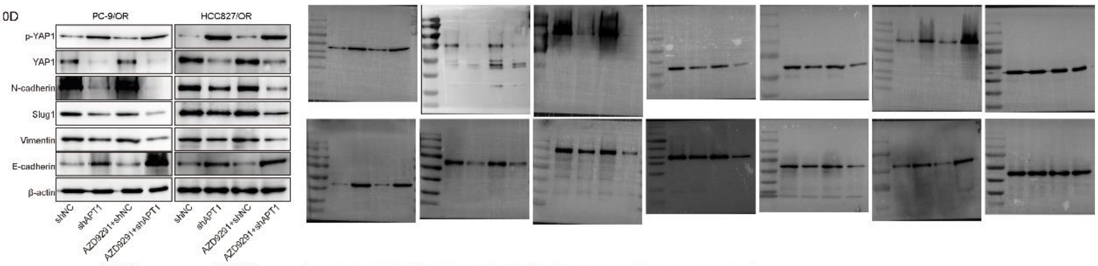

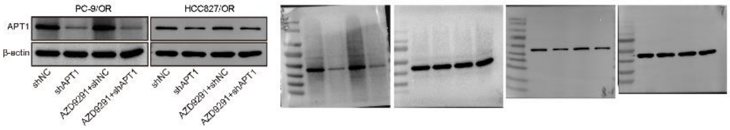

S11A

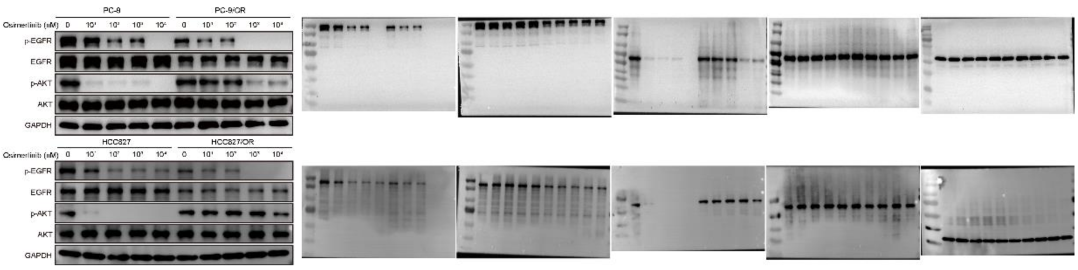

S11B

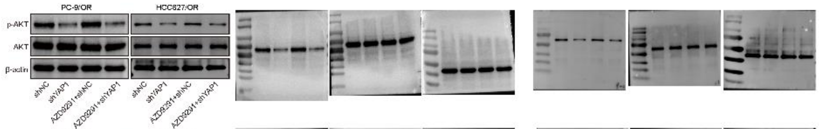

S11C

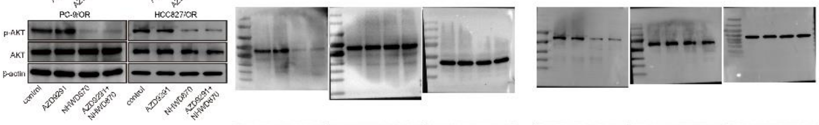

S11D

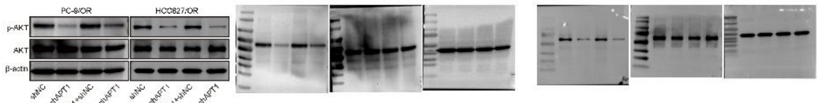

S11E

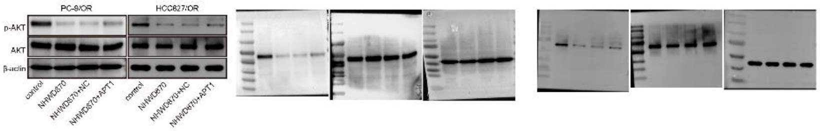

S12D

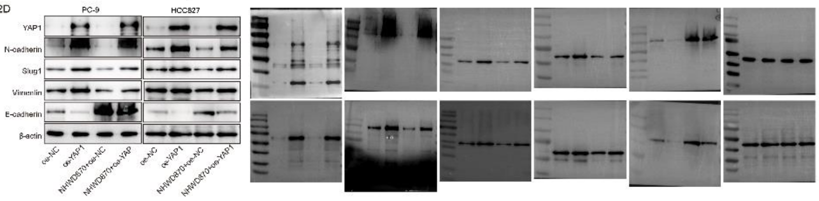

S13A

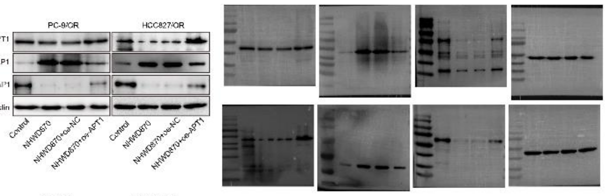

S13C

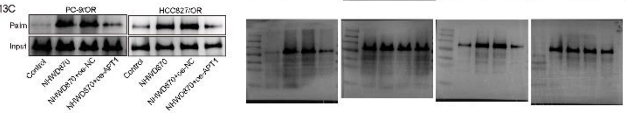

Supplement: Supplementary file 1 — Supplementary Materials [file 41420_2025_2794_MOESM1_ESM.pdf]
